# West African Clade C MERS-CoV Strains BF785 and Mor213 Induce More Robust Interferon and Inflammatory Signaling Compared to Clade A in Human Respiratory Cells

**DOI:** 10.3390/v18090935

**Published:** 2026-08-27

**Authors:** Helen A. Stillwell, Helena Winstone, Cailu Lin, Anant K. Patel, Li Hui Tan, Sunil Singhal, Danielle R. Reed, Noam A. Cohen, Susan R. Weiss, Ranawaka A. P. M. Perera

**Affiliations:** 1 Department of Microbiology, University of Pennsylvania, Philadelphia, PA 19104, USA; helen.stillwell@pennmedicine.upenn.edu (H.A.S.); helena.winstone@pennmedicine.upenn.edu (H.W.); anant.patel@pennmedicine.upenn.edu (A.K.P.); 2 Penn Center for Research on Emerging Viruses, University of Pennsylvania, Philadelphia, PA 19104, USA; 3 Monell Chemical Senses Center, Philadelphia, PA 19104, USA; clin@monell.org (C.L.); reed@monell.org (D.R.R.); ncohen@pennmedicine.upenn.edu (N.A.C.); 4 Corporal Michael J. Crescenz VA Medical Center, Philadelphia, PA 19104, USA; lihtan@pennmedicine.upenn.edu; 5 Division of Thoracic Surgery, Department of Surgery, University of Pennsylvania, Philadelphia, PA 19104, USA; 6 Otorhinolaryngology-Head and Neck Surgery, University of Pennsylvania, Philadelphia, PA 19104, USA

**Keywords:** MERS-CoV, clade C, NS4b, OAS-RNase L, coronaviruses, virus–host interaction, innate immunity

## Abstract

Clade A Middle East respiratory syndrome coronavirus (MERS-CoV) spilled over from camels into humans in Saudi Arabia in 2012 and caused pneumonia and severe respiratory disease, with a 37% case fatality rate. While clade A and B viruses are closely related phylogenetically, clade B MERS-CoV strains have since outcompeted clade A strains and continue to circulate in camels and humans in the Middle East and cause outbreaks in humans. Clade C strains circulate in camels across the African continent but have not been reported to cause disease in humans. We have shown that a number of East African clade C isolates are less able to utilize the TMPRSS2-mediated pathway for viral entry in both cell lines and primary nasal epithelial cultures, which may underlie the reduced replication of East African clade C strains in humans. However, the reduced replicative capacity of West African strains in human cells appears to be independent of viral entry, suggesting an alternative basis for their attenuation. Here, we report that West African clade C MERS-CoV isolates with deletions in *ORF4b* encoding a key accessory protein, NS4b, induced significantly more robust interferon and inflammatory responses than clade A MERS in human respiratory cell lines and primary bronchial air–liquid interface cultures. The replication deficit for the West African strain BF785, which has a complete deletion of *ORF4b*, was partially rescued when *RNASEL* was knocked out in A549 cells. These findings demonstrate that complete loss of NS4b results in stronger innate immune activation than partial truncation and suggests that differential selection on NS4b may contribute to the varying phenotypes of clade C MERS-CoV strains circulating in African camels.

## 1. Introduction

Middle East respiratory syndrome coronavirus (MERS-CoV) was first identified in humans in Saudi Arabia and Jordan in 2012 [1]. MERS-CoV is a merbecovirus within the genus *Betacoronavirus,* the first of its subgenus known to infect humans. Dromedary camels are the principal animal reservoir for MERS-CoV, but phylogenetic evidence suggests that MERS-CoV ancestral strains, like SARS-CoV and SARS-CoV-2, originated in bats [2,3,4,5,6]. In humans, MERS-CoV causes a spectrum of disease, ranging from asymptomatic to severe pneumonia, with a high case fatality ratio of 37% for reported cases [7]. Infected dromedaries, however, present with asymptomatic-to-mild respiratory disease [7]. Due to its pathogenesis in humans, the potential emergence of more transmissible variants, and the lack of a licensed therapeutic or vaccine, MERS-CoV is a World Health Organization (WHO) priority pathogen for research and development [1].

MERS-CoV circulates widely in dromedary camel populations throughout the Middle East and Africa and has also been detected in parts of South and Central Asia [7]. MERS-CoV strains are phylogenetically categorized into three clades—A, B, and C—which cluster by geographic origin [8]. Clade A contains the Erasmus Medical Center (EMC) strain (MERS-CoV EMC/2012), which was isolated from a human during the 2012 outbreak in Saudi Arabia and is considered wild-type virus here (wtMERS) [9]. Clade B contains strains that currently circulate in camels across the Middle East and cause periodic spillover events into humans [9,10]. Clade C viruses circulate in camels in Africa and are further categorized by geographic subclades: West Africa (C1.1), East Africa (C1.2 and C2), and Egypt (C3) [11,12]. These viruses diverge from the clade A and B viruses phylogenetically and contain amino acid substitutions or deletions in the spike protein and the accessory proteins NS3, NS4b, and NS5, in addition to substitutions in other areas of the genome with unconfirmed function [8]. The spike protein mediates viral entry, and some of the accessory proteins have been shown to antagonize innate immune responses [13]. More than 84% of all human cases reported by Saudi Arabia have been due to direct or indirect contact with infected dromedary camels or infected individuals in health-care facilities [7]. Interestingly, no human outbreaks of clade C MERS viruses have been reported on the African continent, despite its widespread prevalence in camels and documented exposure of humans to infected camels [14,15]. There are serological studies that show evidence of human infection in Africa, but most are associated with occupational exposure to camels or travel to the Arabian Peninsula and suggest that zoonotic transmission is rare [16,17,18,19,20,21,22,23,24]. Other studies have found MERS-CoV-specific T cells in dromedary-exposed workers in Africa in addition to PCR-positive asymptomatic cases [25,26,27]. Additionally, camelids in Mongolia, Australia, and parts of Asia and America do not harbor MERS-CoV but present a naïve population that could be infected with potential spillover into humans [28,29].

Clade C MERS-CoV strains have been shown to be attenuated for replication compared to wild-type MERS-CoV in human cells [8,11,30]. Recently, we reported that the spike proteins of clade C isolates are less well-cleaved at the S1/S2 boundary than clade A or B viral spike proteins, and that three East African clade C strains are less able to utilize the TMPRSS2-mediated entry pathway in cell lines and primary epithelial cultures [30]. While the reduced usage of the TMPRSS2-mediated entry pathway may underlie the replication deficit we observe with the East African clade C strains in human cells, the replication deficit for the West African clade C strains appears to be independent of viral entry. Following viral entry, the innate immune system is the next line of defense against viral replication and spread. Here, we sought to determine whether the amino acid substitutions or deletions present in West African clade C strains in key accessory genes known to antagonize innate immunity might account for these differences we observe in viral replication in human cells.

The 3′ one-third of the human coronavirus (HCoV) genome contains the accessory genes [31]. The HCoV accessory proteins contribute to the unique characteristics of each virus, and most antagonize innate immune responses. MERS-CoVs encode the accessory proteins NS3, NS4a, NS4b, NS5, and NS8b (encoded by *ORF3*, *ORF4a*, *ORF4b*, *ORF5*, and *ORF8b*) [32,33,34]. The West African clade C viral isolates Nig1657, Mor213, and BF785 all contain deletions of varying size in *ORF4b*, encoding a well-described innate immune antagonist [8]. NS4b has two known functional domains. The first contains a phosphodiesterase (PDE) that prevents activation of the oligoadenylate synthetase–ribonuclease L (OAS-RNase L) antiviral pathway and weakly inhibits interferon (IFN) signaling [35,36,37,38,39]. Upon sensing dsRNA, oligoadenylate synthetases (OAS1-3) synthesize 2-5A, which promote the cleavage of viral and host RNA through the activation of the host enzyme ribonuclease (RNase) L, an endoribonuclease that cleaves viral and cellular ssRNA [40,41]. The NS4b PDE functions to degrade 2′,5′-oligoadenylates (2-5A), thereby preventing activation of RNase L. The second is an amino-terminal nuclear localization signal (NLS), which has been reported to block NF-κB translocation to the nucleus downstream of dsRNA sensing by MDA5, thereby limiting transcriptional upregulation of type I and type III IFNs and other proinflammatory cytokines [8,35,42,43,44]. MERS-CoV NS3, NS4a, and NS5 have also been implicated in antagonizing that innate immune response. NS4a is a double-stranded RNA binding protein that prevents ds-RNA sensing upstream of the IFN response, namely the PKR pathway, and OAS-RNase L [37]. A MERS-CoV mutant with an *ORF3-5* deletion showed increased IFN and inflammation-response induction [45]. Additionally, deletion of *ORF5* suggested a partial role for the NS5 protein in NF-kκB-mediated inflammation [45]. As such, NS4b, in conjunction with the other MERS accessory proteins, dampens the innate immune response and allows early infection to progress with minimal detection [46].

**Figure 1:**
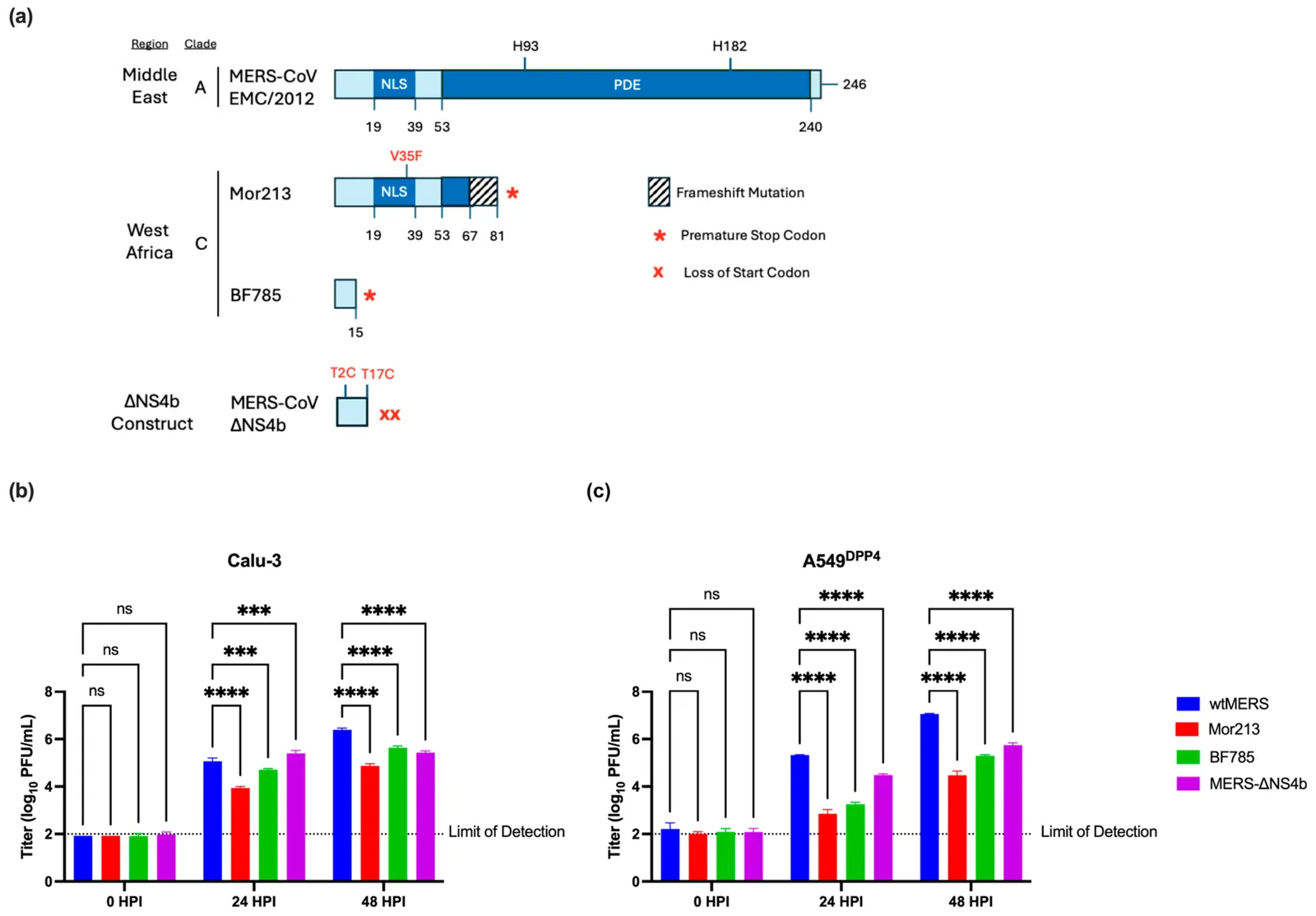
(**a**) Schematic of changes sustained to NS4b in the clade C viruses and MERS-ΔNS4b compared to the full-length protein in wtMERS. The amino acid substitutions T2C and T17C in MERS-ΔNS4b are made in the overlap region between NS4a and NS4b to abrogate putative start codons, effectively preventing expression of the protein from the second amino acid. The catalytic histidine residues H93 and H182 are indicated in the full-length clade A PDE domain. Calu-3 (**b**) or A549^DPP4^ (**c**) cell lines were infected for 24 and 48 hpi (MOI = 0.1 PFU/cell), and infection was quantified by plaque assay. Data are from one representative of three independent experiments. Data are displayed as means ± SD. Statistical significance compared to wtMERS was calculated by two-way ANOVA: *** *p* = 0.0003 and **** *p* ≤ 0.0001.

Here, we utilize a panel of four viruses—wild-type (wt)MERS (MERS-CoV EMC/2012); two clade C West African strains, Mor213 and BF785; and clade A-derived MERS-ΔNS4b mutant (Figure 1a)—to determine whether the expression of a truncated NS4b protein or loss of expression of NS4b protein during infection with these West African camel strains contributes to their attenuation in human cells. The construction of MERS-ΔNS4b has been previously described and serves as a positive control for complete absence of NS4b expression [35,47]. Mor213 expresses an NS4b protein with an intact NLS sequence (containing a V35F substitution whose functional impact is unknown) and a truncation of the PDE domain (including both catalytic HIS residues), while BF785 expresses an NS4b with complete deletion of both the NLS and PDE domains. Compared to the MERS-CoV EMC/2012 spike protein, Mor213 and BF785 contain seven and five amino acid substitutions, respectively; however, none of these substitutions is in direct receptor-contacting residues or the S1/S2 cleavage site, and we did not observe significant defects in viral entry for these viruses [30]. We did not include the West African virus Nig1657 in this panel because it also has a deletion in the NS3 protein [8]. Mor213 and BF785 have some amino acid substitutions in NS3 that are yet to be characterized. Additionally, both viruses contain amino acid substitutions in NS5; however, these are conserved across clade C strains. We show that BF785 induces a more robust IFN and proinflammatory cytokine response than wtMERS comparable to the MERS-ΔNS4b mutant in a human respiratory cell line. Mor213 behaves similarly to wtMERS and induces little IFN and proinflammatory cytokines at the RNA level, likely because its NS4b retains the NLS. Interestingly, we also show that infection of primary human bronchial air–liquid interface (ALI) cultures with Mor213 and BF785 recapitulates the replication defect and immunostimulatory phenotype observed in human cell lines in a donor-dependent manner. Furthermore, RNase L is activated following infection with BF785 and, to a lesser extent, Mor213 in a human respiratory cell line, and infecting *RNASEL* knockout (KO) cells partially recovers the replication deficit for BF785. Ultimately, we suggest that the differences in the extent to which these viruses antagonize the innate immune response may contribute to the lack of spillover of clade C viruses into humans in West Africa and point to differential selection pressures on NS4b between MERS-CoV strains circulating in camels.

## 2. Materials and Methods

### 2.1. Cell Lines and Plasmids

Calu-3 (ATCC) and Vero-CCL81 (ATCC) were cultured in Dulbecco’s Modified Eagle Medium (DMEM) (Gibco, Waltham, MA, USA) supplemented with 10% fetal bovine serum (FBS) and 1 X penicillin/streptomycin (Gibco, Waltham, MA, USA) and incubated at 37 °C and 5% CO_2_. A549^DPP4^, A549^DPP4^ RNase L KO, A549^DPP4^ PKR KO, and A549^DPP4^ MAVS KO cells were constructed from A549 background cells (ATCC) overexpressing the MERS-CoV receptor dipeptidyl peptidase-4 (DPP4), as previously described [37,38], and cultured in RPMI 1640 (Gibco, Waltham, MA, USA) supplemented with 10% FBS and penicillin/streptomycin (Gibco, Waltham, MA, USA) and incubated at 37 °C and 5% CO_2_.

### 2.2. Primary Bronchial ALI Cultures

Primary bronchial cells were obtained from noncancerous regions during lung resections for cancer treatment from patients (all de-identified) in the Department of Otorhinolaryngology—Head and Neck Surgery, Division of Rhinology at the University of Pennsylvania and the Philadelphia Veteran Affairs Medical Center, after obtaining informed consent. The protocol was approved by the University of Pennsylvania Institutional Review Board (protocol #813004). Patients with a history of systemic disease or on immunosuppressive medications were excluded. ALI cultures were grown and differentiated on 0.4 µm pore Transwell inserts. Specimens were dissociated and fibroblasts removed before plating onto Transwell inserts. Bronchial cells were allowed to grow to confluence in the submerged state for approximately 5 days and then differentiated with basal differentiation media. Cultures were differentiated for 4 weeks prior to infection. PneumaCult™-Ex Plus Medium was used for cell expansion, and PneumaCult-ALI basal medium (Stemcell Technologies, Cambridge, MA, USA) was used for all experiments.

### 2.3. Viruses

The following viruses were used in this study: MERS-CoV EMC/2012 (wtMERS-CoV) (GenBank ID: NC_019843.3), Mor213 (GenBank ID: MG923469.1), and BF785 (GenBank ID: MG923471.1) were obtained from Malik Peiris, School of Public Health, the University of Hong Kong. MERS-CoV-ΔNS4b was obtained from Ralph Baric, University of North Carolina, and constructed as described previously [35,47]. Viral stocks were generated and titered by plaque assay in Vero-CCL81 cells. Viral genomes were sequenced by Azenta and the following amino acid substitutions found relative to the reference sequences: MERS-CoV EMC/2012—ORF1a (S1965F, nsp3) and Mor213—N (K384N). No changes were identified in NS4a or other regions expected to broadly impact dsRNA sensing.

### 2.4. Infections and Viral Titers

All infections were conducted in a biosafety level 3 laboratory using procedures approved by the University of Pennsylvania Institutional Biosafety Committee, and with appropriate personal protective equipment and protocols. For cell-line infections, viruses were diluted in serum-free DMEM (Calu-3) or RPMI (A549^DPP4^) to MOI = 0.1 PFU/cell and added to cells for adsorption for 1 h at 37 °C. Cells were then washed three times with phosphate-buffered saline (PBS) and re-supplied with DMEM + 2% FBS for Calu-3 or RPMI + 2% FBS for A549^DPP4^. At the indicated times, 100 μL of supernatant was collected and stored at −80 °C for titration by plaque assay on Vero CCL81 cells. For primary bronchial ALI infections, viruses were diluted in serum-free DMEM to MOI 0.1 PFU/cell in a total inoculum volume of 50 μL. Inoculum was added to the apical compartment of each Transwell for adsorption for 1 h at 37 °C. Cells were then washed three times with PBS, and a fourth PBS wash was collected to confirm sufficient removal of input virus and serve as the 0 h post-infection (hpi) timepoint for titers. At the indicated times after infection, airway surface liquid (ASL) was collected by the addition of 200 μL of PBS to the apical compartment of each well. ASL samples were stored at −80 °C for titration by plaque assay on Vero-CCL81 cells. wtMERS and MERS-ΔNS4b were titered by plaque assay with liquid overlay (DMEM with 2% FBS, 1× sodium pyruvate, and 0.1% agarose) for 3 days. Mor213 and BF785 were titered similarly, except with overlay containing 0.15% agarose, and for 4 days in the case of Mor 213. Plaques were fixed in 4% paraformaldehyde (PFA) for 30 min at room temperature, removed from the BSL-3, and stained with 0.1% Crystal Violet.

### 2.5. Reverse Transcriptase (RT)–Quantitative PCR

Cells were lysed at indicated timepoints with buffer RLT Plus (Qiagen, Germantown, MD, USA), and RNA was extracted according to the manufacturer’s protocol. RNA was then reverse-transcribed to produce cDNA using a High-Capacity cDNA Reverse Transcriptase kit (Applied Biosystems, ThermoFisher, Waltham, MA, USA). This cDNA was amplified using specific RT-qPCR primers for each target gene (primer sequences are listed in Table A1). Fold changes in mRNA levels for indicated interferons (IFNs), interferon-stimulated genes (ISGs), and cytokines were reported as fold changes over mock-infected cultures using the formula 2^−Δ(ΔCt)^.

### 2.6. Western Blotting

Cell lysates were harvested at indicated timepoints using RIPA buffer (50 mM Tris pH 8, 150 mM NaCl, 0.5% deoxycholate, 0.1% SDS, and 1% NP40) supplemented with protease inhibitors (Roche, Indianapolis, IN, USA) and phosphate inhibitors (Roche). Lysates were mixed 1:1 with 2X Laemmli sample buffer (Bio-Rad, Hercules, CA, USA), boiled at 95 °C for 10 min, and then separated via sodium dodecyl sulfate–polyacrylamide gel electrophoresis (SDS/PAGE) and transferred to polyvinylidene difluoride (PVDF) membranes. Membranes were blocked in 5% bovine serum albumin (BSA) or 5% non-fat milk before being probed with the primary antibodies listed in Table A2. Membranes were then washed and probed with the secondary antibodies listed in Table A3. Proteins were detected using SuperSignal West Femto Substrate (34095, Thermo Fisher, Waltham, MA, USA) or SuperSignal West Pico PLUS Chemiluminescent Substrate (34580, Thermo Fisher, Waltham, MA, USA) on an ImageQuant Amersham 680. If needed, blots were stripped using Thermo Scientific Restore Western Blot stripping buffer (catalog #21059) for 30 min at room temperature before being re-probed.

### 2.7. Bulk RNA Sequencing and Analysis

Calu-3 cells were infected at MOI = 0.1 PFU/cell with the indicated viruses or mock-infected (n = 3, one replicate from three independent experiments). Cells were lysed at 24 hpi using RLT Plus buffer, and total RNA was extracted using Qiagen RNeasy Plus Mini kit (Cat. No. 74004). Samples were sequenced by Genewiz (South Plainfield, NJ, USA), with a sequencing depth of 30 million reads per sample. RNA-sequencing libraries were prepared using the NEBNext Ultra II RNA library Prep Kit for Illumina, using the manufacturer’s instructions (NEB, Ipswich, MA, USA). Briefly, mRNAs were initially enriched with Oligod(T) beads. Enriched mRNAs were fragmented for 15 min at 94 °C. First-strand and second-strand cDNA were subsequently synthesized. cDNA fragments were end-repaired and adenylated at 3′ends, and universal adaptors were ligated to cDNA fragments, followed by index addition and library enrichment by PCR with limited cycles. The sequencing library was validated on the Agilent TapeStation (Agilent Technologies, Palo Alto, CA, USA) and quantified by Qubit 3.0 Fluorometer (Invitrogen, Carlsbad, CA, USA), as well as by quantitative PCR (KAPA Biosystems, Wilmington, MA, USA). The sequencing libraries were multiplexed and clustered onto a flow cell on the Illumina NovaSeq Xplus instrument according to the manufacturer’s instructions. The samples were sequenced using the 2 × 150 bp paired-end (PE) configuration. Image analysis and base calling were conducted by the NovaSeq Control Software (NCS, v1.3.1). Raw sequence data (.bcl filed) generated from Illumina NovaSeq were converted into fastq files and de-multiplexed using Illumina bcl2fastq 2.20 software. One mismatch was allowed for index sequence identification. Raw paired-end sequencing reads were trimmed using Trim Galore v0.6.10 (Cutadapt v4.4). Trimmed reads were quantified against the Homo sapiens GRCh38 (hg38) reference transcriptome using Salmon v1.10.2 with quasi-mapping. Transcript abundances were summarized to gene-level counts for downstream analyses. Log_2_ fold-change values calculated for IFN response genes were annotated from a modified Molecular Signatures Database (MSigDB), HALLMARK_INTERFERON_ALPHA_RESPONSE [48].

### 2.8. Immunofluorescence (IF) Staining

A549^DPP4^ and A549^DPP4^ RNase L KO cells were seeded on glass-bottom plates (P24-1.5H-N, Cellvis, Mountain View, CA, USA). Cells were infected at MOI 0.5 PFU/cell. One hour post-adsorption, cells were washed three times, re-supplied with 2% media, and incubated at 37 °C 5% CO_2_ for 24 h. At 24 hpi, cells were fixed with 4% PFA for 30 min at room temperature and removed from the BSL-3. Cells were washed with PBS and permeabilized with 0.1% Triton X-100 in PBS for 15 min at room temperature, washed with PBS, and subsequently blocked with 3% BSA in PBS for 30 min at room temperature. Cells were incubated with anti-nucleocapsid (40068-MM10, Sino Biological, Houston, TX, USA) or anti-PABPC1 (AB21060, Abcam, Waltham, MA, USA) 1:1000 in 3% BSA overnight at 4 °C, with rocking. Cells were washed 3 times with PBS and incubated with secondary Alexa Fluor antibodies in 3% BSA for 1 h at room temperature, with rocking. Cells were washed 3 times, and DAPI in PBS was added for 5 min at room temperature. DAPI was washed off with PBS, and images were acquired on a Nikon Ti2 Eclipse (Nikon Corporation, Tokyo, Japan) at 20× magnification. Six images per condition were acquired.

## 3. Results

### 3.1. West African Clade C Isolates Expressing Mutant NS4b Show Replication Defect Compared to Wild-Type MERS in Human Lung-Derived Epithelial Cell Lines

To begin to determine the post-entry factors impacting the replication of West African clade C strains in human cells, we utilized two camel isolates, Mor213 and BF785, which contain unique deletions in *ORF4b*—the gene encoding NS4b, a well-characterized innate immune antagonist [11,35,37,38]. The genomes of these viruses do not possess deletions in any other accessory genes and therefore can be used to determine the effects of the deficits in the NS4b protein that have been selected for in camels in West Africa over time. NS4b of Mor213 contains the amino acid substitution V35F in the NLS, a frameshift mutation at amino acid 67, and a premature stop codon at amino acid 81, resulting in a loss of the PDE (Figure 1a). BF785 encodes a truncated NS4b with complete deletion of both the NLS and PDE due to a premature stop codon at amino acid 15 (Figure 1a). MERS-ΔNS4b encodes mutations in the overlapping region between NS4a and NS4b to abrogate putative start codons (T2C and T17C), effectively truncating the protein at the second amino acid (Figure 1a). Premature stop codons were inserted at A91T, C97A, and C102G, and residues 106–669 were deleted to remove the majority of NS4b but leave transcriptional regulatory sequence 5 in place (Figure 1a) [35,47]. We first confirmed the replication kinetics of this panel of viruses in human lung-derived cell lines. Calu-3 cells (Figure 1b) and A549^DPP4^ (Figure 1c) were infected at a multiplicity of infection (MOI) of 0.1 PFU/cell with wtMERS, Mor213, BF785, and MERS-ΔNS4b, and supernatant samples were harvested at 0, 24, and 48 h post-infection (hpi).

Quantification of infectious virus was completed by plaque assay in the Vero CCL81 cell line. In three independent experiments, both camel-derived isolates were attenuated for replication compared to wtMERS at both timepoints, consistent with the literature [8,11,30]. Mor213 is the most attenuated, with replication reduced by 2 log_10_ at peak replication in both cell types. At 48 hpi, the MERS-ΔNS4b mutant was also attenuated compared to wtMERS at and showed similar replication kinetics to BF785.

### 3.2. West African Clade C Virus BF785 Induces More Robust IFN, ISG, and Inflammatory Marker Expression Relative to Wild-Type MERS

Next, we sought to determine whether the activation of the type I/III IFN pathways was increased during infection with Mor213 and BF785. wtMERS has been shown to strongly inhibit IFN production in human cells [42,49,50,51]. Typically, upon detection of viral infection, NF-κB translocates to the nucleus and promotes the transcription of IFNs, pro-inflammatory cytokines, and chemokines [42]. It has been shown that the MERS NS4b NLS outcompetes NF-κB for karyopherin-α4 (importin-α3) binding and translocation into the nucleus as a mechanism of interference with the NF-κB-mediated innate immune response [42]. Given that the NLS is retained in Mor213, we hypothesized that it might behave like wtMERS and antagonize IFN and inflammatory cytokine expression upon infection. However, it does contain an amino acid substitution (V35F) in the NLS that has unknown impact on function. Additionally, we expected BF785 would fail to antagonize NF-kB-dependent innate immune responses similar to MERS-ΔNS4b due to its loss of the NLS [11]. Indeed, BF785 has shown increased IFN and inflammatory cytokine expression in human cells compared to wtMERS [8].

**Figure 2:**
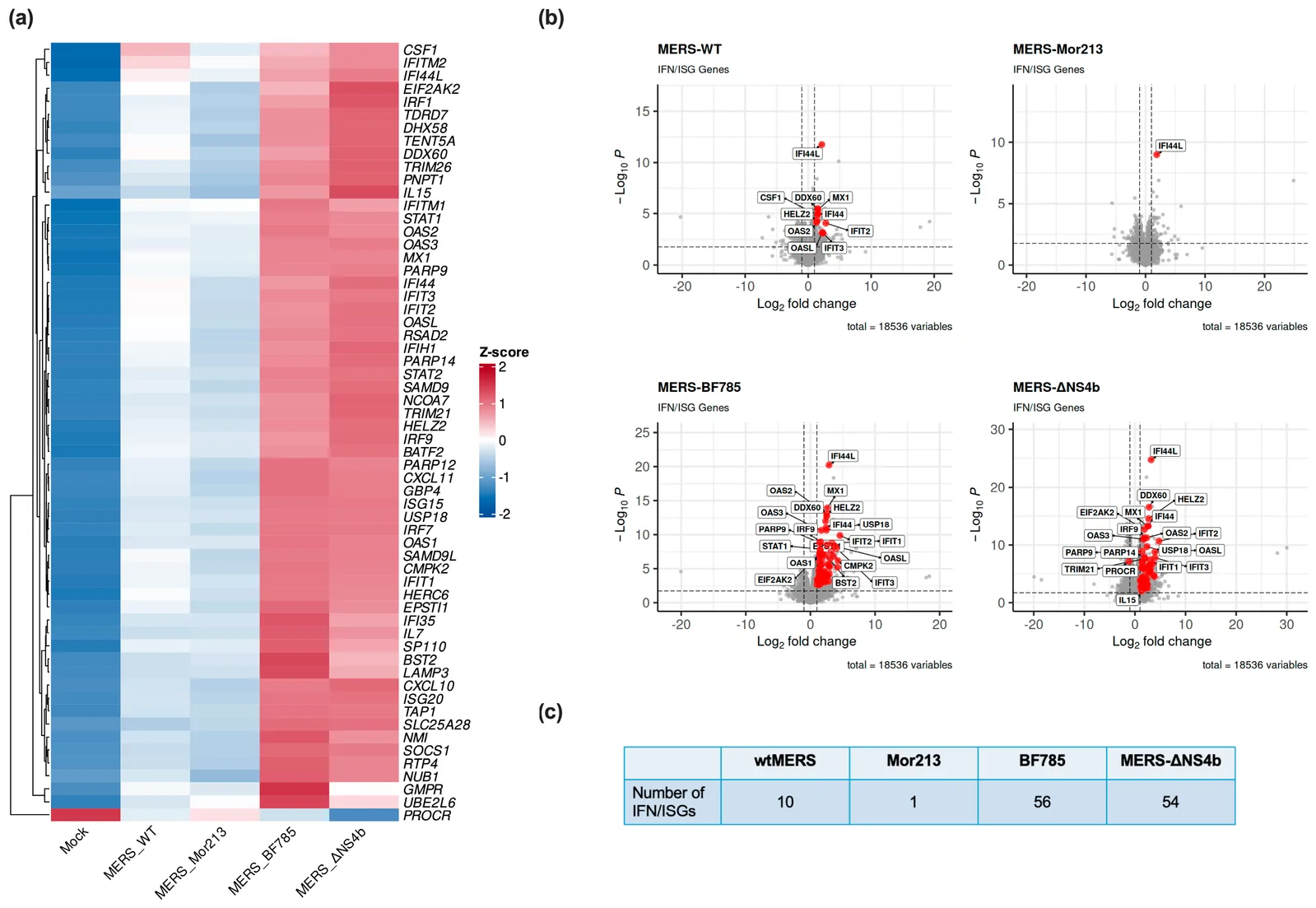
Calu-3 cells were infected with wtMERS, Mor213, BF785, and MERS-ΔNS4b (MOI = 0.1 PFU/cell); they were lysed at 24 hpi; and their RNA was extracted and analyzed by RNA-seq. (**a**) Heatmap was generated via hierarchical clustering of IFN-related genes. Viruses were ranked in terms of degree of induction of each ISG based on log_2_ fold-change values, from least (blue) to most upregulation (red). (**b**) Volcano plots for differentially expressed genes for each virus relative to mock-infected cultures. Genes involved in the IFN signaling response are indicated in red. Significance cutoffs are indicated by dashed lines for both log_2_ fold-change values and adjusted *p*-values (padj). (**c**) The total number of ISGs reaching significance thresholds for each virus was quantified.

To test this, we infected Calu-3 cells at an MOI of 0.1 PFU/cell with all four viruses, collected total intracellular RNA at 24 hpi, and performed bulk RNA sequencing to identify differentially regulated genes (relative to mock-infected cultures) (Figure 2). Trimmed sequencing reads were quantified against the Homo sapiens GRCh38 (hg38) reference transcriptome using Salmon v1.10.2 with quasi-mapping, and transcript abundances were summarized to gene-level counts. Downstream analyses identified antiviral immune response genes and the IFN response as the most significantly upregulated pathway following infection with BF785 and MERS-ΔNS4b, while, as expected, wtMERS and Mor213 showed little induction of these pathways.

Log_2_ fold-change values calculated for IFN response genes annotated from a modified Molecular Signatures Database (MsigDB) HALLMARK_INTERFERON_ALPHA_RESPONSE gene were combined from each infection and visualized as a heatmap [48] (Figure 2a). We performed hierarchical clustering of IFN response genes to generate the heatmap, ranking log_2_ fold-change values of each gene from least (blue) to most (red) upregulated during infection with all four viruses. For comparison, we included a mock-infected condition. Volcano plots depicting fold changes in normalized transcript abundances relative to mock-infected cultures are shown in Figure 2b. The total number of IFN response genes that reached significance thresholds for each virus is quantified in Figure 2c.

Infection with BF785 and MERS-ΔNS4b induced a larger number of ISGs with greater log_2_ fold-change values over mock-infected cells compared to wtMERS-CoV or Mor213 infection. Mor213 showed very little induction, even less than wtMERS-CoV, suggesting that the NLS domains retain functionality despite the amino acid substitution from valine to phenylalanine at position 91. Additionally, Mor213 may not induce as much expression, given its replication was the most attenuated of the virus panel in Calu-3 cells. Sequencing data for each IFN response gene for our modified MsigDB list (including log_2_ fold change and *p*-values) for each virus infection can be accessed in the Supplementary Materials.

**Figure 3:**
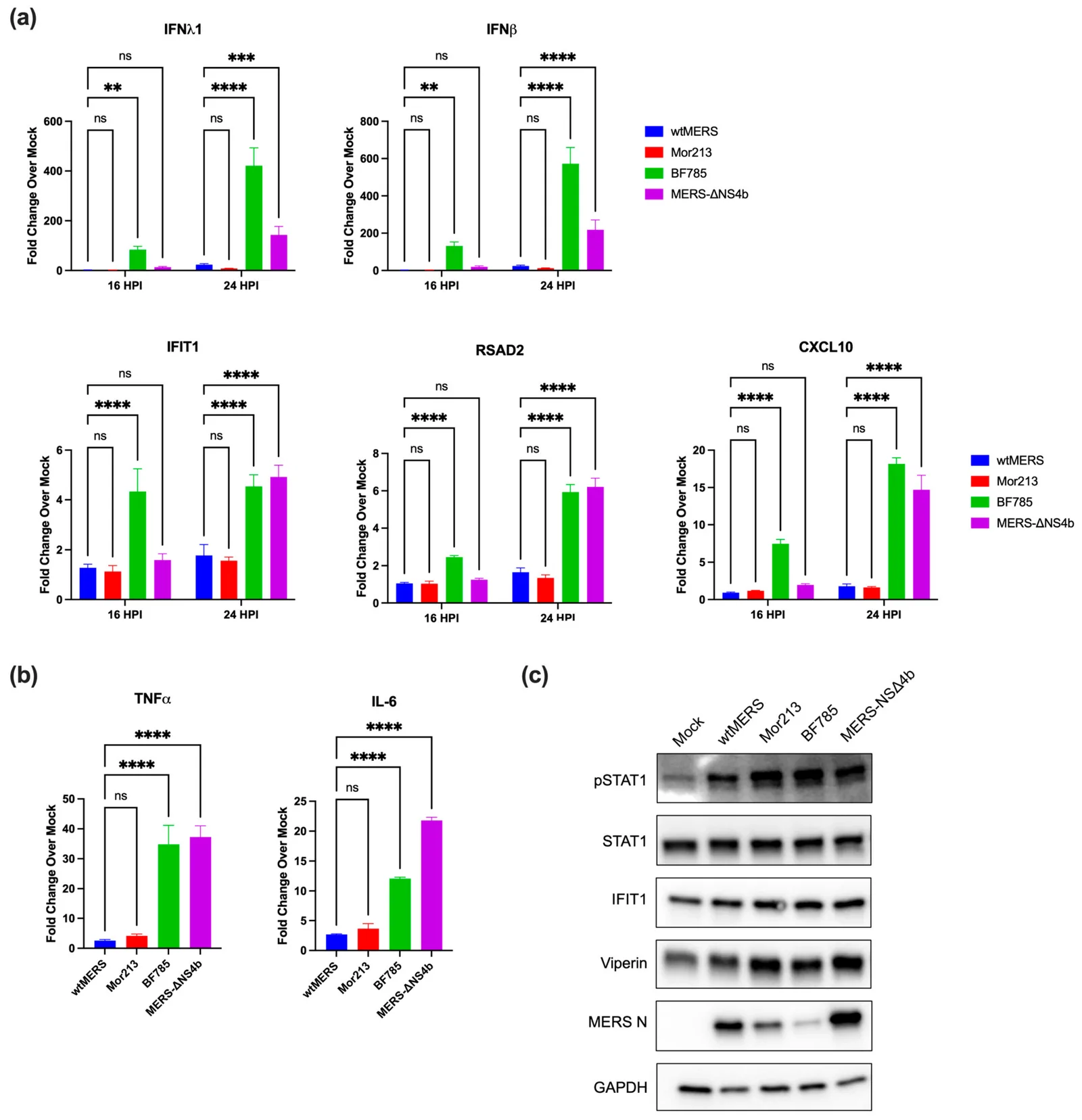
Calu-3 cells were mock-infected or infected (MOI = 0.1 PFU/cell, n = 3). (**a**) Intracellular RNA was harvested at 16 and 24 hpi, and expression of IFNL1, IFNB, IFIT1, RSAD2, and CXCL10 mRNAs was quantified by RT-qPCR and expressed as fold change over mock-infected, using the 2^−Δ(ΔCT)^ formula. Data are from one representative of three independent experiments. (**b**) Calu-3 cells were mock-infected or infected (MOI = 0.1 PFU/cell, n = 3). Total RNA was harvested at 24 hpi, and expression of TNFα and IL-6 mRNAs was quantified by RT-qPCR and expressed as fold change over mock-infected, using the 2^−Δ(ΔCT)^ formula. Data are from one representative of three independent experiments. Data are displayed as means ± SD. Statistical significance compared to wtMERS was calculated by one-way ANOVA: ** *p* ≤ 0.01, *** *p* = 0.0003, and **** *p* ≤ 0.0001. Data that were not statistically significant are not labeled. (**c**) Western blot analysis of whole-cell lysates collected at indicated times following infection. Samples were separated via SDS-PAGE, followed by transfer onto a PVDF membrane for detection using indicated antibodies.

To confirm the RNA-seq data, we infected Calu-3 cells at an MOI of 0.1 PFU/cell with all four viruses and collected total intracellular RNA at 16 and 24 hpi (Figure 3a). We used RT-qPCR to quantify mRNA expression of select IFN genes (IFNL1 and IFNB) and ISGs (IFIT1, RSAD2, and CXCL10), as compared to mock-infected cells. Earlier timepoints were chosen, as there was too much cell death at 48 hpi for BF785 and MERS-ΔNS4b to collect sufficient RNA. As expected, we found minimal expression of IFNs or ISGs compared to mock-infected cells with wtMERS and Mor213. In contrast, IFN mRNAs and ISG mRNAs were significantly induced in BF785 and MERS-ΔNS4b-infected cells compared to wtMERS infection at both 16 and 24 hpi. We also quantified the expression of mRNA encoding the cytokines TNFα and IL-6 upon infection with the same four viruses at 24 hpi (Figure 3b). Inflammatory marker mRNA expression (TNFα and IL-6) was significantly greater in BF785- and MERS-ΔNS4b-infected cells compared to wtMERS or Mor213 infection. Interestingly, BF785- and MERS-ΔNS4b-infected cells showed comparable levels of TNFα, while MERS-ΔNS4b showed a little less than ten-fold more IL-6 than BF785.

The comparison of ISG expression at the RNA level was complemented by examining protein levels. Protein lysates from Calu-3 cells infected with each virus or mock-infected were collected at 24 hpi and analyzed by Western blot to compare levels of STAT1 phosphorylation, as well as the abundance of the ISG-encoded proteins interferon-induced protein with tetratricopeptide repeats 1 (IFIT1) and Viperin (Figure 3c). Immunoblotting for the MERS-CoV nucleocapsid (N) confirmed productive infection and recapitulated the viral titer data. Immunoblotting for glyceraldehyde-3-phosphate dehydrogenase (GAPDH) served as a protein loading control. We observed upregulation of p-STAT1, IFIT1, and Viperin at the protein level for BF785 and MERS-ΔNS4b by Western blot. Surprisingly, we also observed upregulation of p-STAT1, IFIT1, and Viperin for Mor213 in contrast to the lack of ISG induction observed by RT-qPCR and RNA-seq.

### 3.3. Infection of Primary Human Bronchial Air–Liquid Interface Cultures with wtMERS and Clade C Viruses Reproduces Attenuation and Immunostimulatory Phenotype, Albeit in a Donor-Dependent Manner

**Figure 4:**
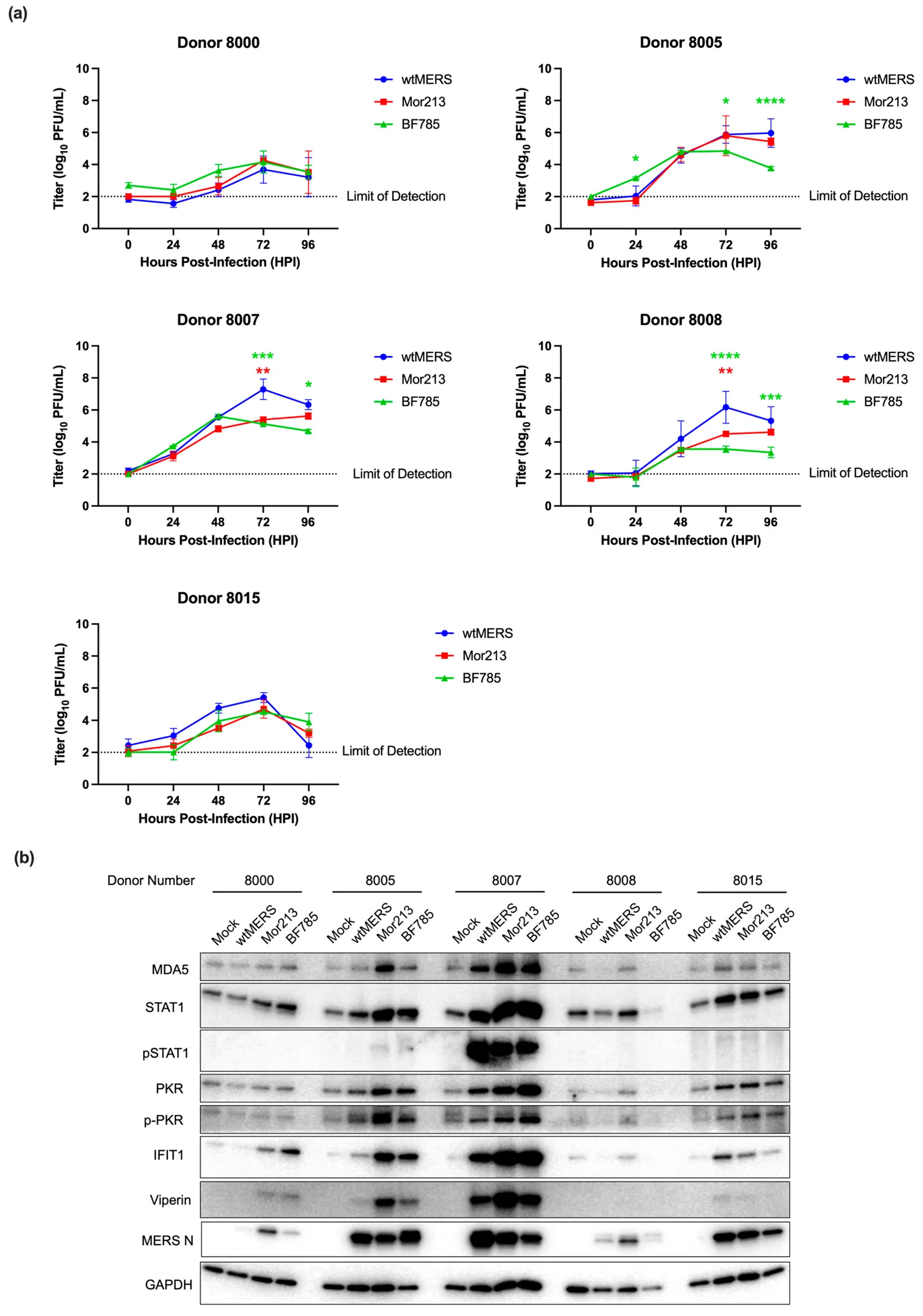
(**a**) Individual donor bronchial ALI cultures were infected at the apical surface in triplicate with the indicated viruses (MOI = 0.1 PFU/cell, 37 °C). Apical surface liquid (ASL) was collected at the indicated timepoints post-infection and quantified by plaque assay. Averaged titers from infections in 5 independent donors infected in triplicate are shown. Data are displayed as means ± SD. Statistical significance compared to wtMERS was calculated by two-way ANOVA: * *p* ≤ 0.05, ** *p* ≤ 0.01, *** *p* = 0.0003, and **** *p* ≤ 0.0001. Data that were not statistically significant are not labeled. (**b**) Western blot analysis of whole-cell lysates collected at 96 hpi. Samples were separated via SDS-PAGE, followed by transfer onto a PVDF membrane for detection using indicated antibodies.

While A549^DPP4^ and Calu-3 cells are lung-derived epithelial cell lines, we wanted to confirm if differences in replication and innate immune activation were detectable in primary human cells. In severe cases, MERS causes lower respiratory disease, so we modeled that cellular tropism by using primary human bronchial cells cultured at air–liquid interface (ALI) to recapitulate the lower airway. Ciliated cells are more abundant in the upper respiratory tract, while goblet cells, which are infected by MERS-CoV, are more abundant in the lower respiratory tract, consistent with the lower respiratory disease observed with MERS-CoV infection [13,52]. ALI Cultures from five healthy donors (Donor IDs 8000, 8005, 8007, 8008, 8015) were infected with wtMERS, Mor213, and BF785 at the apical surface (MOI = 0.1 PFU/cell, n = 3). The MERS-ΔNS4b control was not used in this experiment because we were only able to obtain a limited number of cells for each donor. Apical surface liquid (ASL) was collected at 0, 24, 48, 72, and 96 hpi and titered by plaque assay to characterize viral replication kinetics in bronchial cultures (Figure 4a). Average shed viral titers over time for each virus are shown by donor in Figure 4a.

The viral growth curves separated into two kinetic patterns—in accordance with the cell line data, three (8005, 8007, and 8008) of the five donors replicated wtMERS to higher titer than the camel isolates, Mor213 and BF785, at select timepoints, with BF785 showing greater attenuation than Mor213, on average. However, the two remaining donors (8000 and 8015) showed relatively poor and comparable viral titers for all three viruses. Previously, we showed that primary human nasal ALI cultures infected with wtMERS also exhibited donor-to-donor variability, resulting in a bimodal replication phenotype [53]. Indeed, while nasal cultures from some donors were highly susceptible to wtMERS infection, other donors showed minimal wtMERS replication [53]. Clinically, MERS presents with a spectrum of disease from asymptomatic to severe lower respiratory symptomology, so perhaps the differential viral replication we observe between donors reflects this spectrum of disease and genetic differences in innate responses.

Protein lysates were also collected from bronchial cultures infected with wtMERS, Mor213, and BF785 and mock-infected cultures at 96 hpi and analyzed by Western blot to compare levels of STAT1 phosphorylation and ISG abundance at the protein level (MDA5, phosphorylated-PKR, PKR, IFIT1, and Viperin) (Figure 4b). As in Figure 3c, immunoblotting for the MERS-CoV N confirmed infection and mirrored the titer data, and immunoblotting for GAPDH served as a protein loading control. Interestingly, each donor shows a unique signature of ISG induction. When targets are normalized to GAPDH (Supplementary Figure S1), donors 8005 and 8007, and, to a lesser extent, 8000, Mor213, and BF785, exhibited more robust ISG induction than wtMERS, on average.

### 3.4. OAS-RNase L Pathway Is Activated Following Infection with Mor213, BF785, and MERS-ΔNS4b Mutant, but Not wtMERS

**Figure 5:**
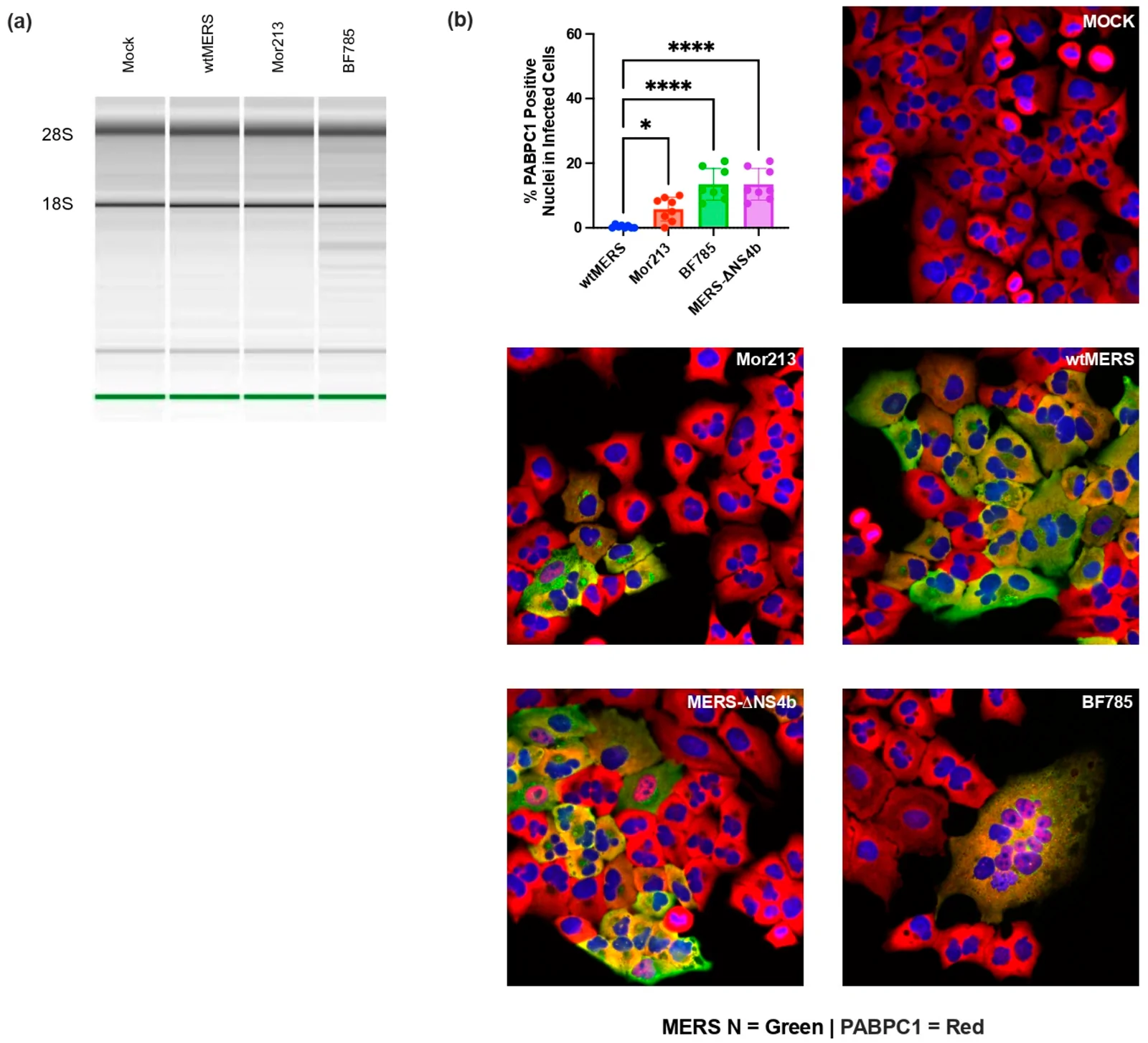
(**a**) A549^DPP4^ wild-type cells were mock-infected or infected at MOI 0.1 PFU/cell, and total cellular RNA was harvested at 24 hpi. The rRNA degradation was assessed on an Agilent Bioanalyzer. 28S and 18S positions are indicated. Data are from one representative of three independent experiments. (**b**) A549^DPP4^ cells were infected in duplicate at MOI = 0.5 PFU/cell with wtMERS, Mor213, BF785, and MERS-ΔNS4b; fixed 24 hpi in 4% PFA; and stained for MERS N (green), PABPC1 (red), and DAPI (blue). Images were acquired on a Nikon Eclipse Ti 2, and the percentage of nuclei in infected cells or syncytia (cells positive for MERS N) with nuclear PABPC1 was determined. Eight fields of view were analyzed per condition. All experiments are n = 3 in technical duplicate, with 6 images taken per condition per experiment. Data are displayed as means ± SD. Statistical significance compared to wtMERS was calculated by one-way ANOVA: * *p* ≤ 0.05 and **** *p* ≤ 0.0001. Uncropped images separated by channel appear in Supplementary Figure S2.

We next investigated whether the OAS-RNase L pathway was activated during Mor213 or BF785 infection. OAS1, -2, and -3 isoforms produce 2-5A in response to viral dsRNA to activate RNase L, which cleaves viral and host ssRNA to restrict viral replication and spread [37,40,41]. It is known that wtMERS antagonizes the activation of RNase L via the NS4b PDE, which degrades 2-5A, preventing activation of RNase L [35,37]. We hypothesized that Mor213 and BF785 would activate RNase L given that the PDE is compromised in NS4b of both strains. To test this, we infected A549^DPP4^ cells with all four viruses (MOI = 0.1 PFU/cell, n = 3), collected total cellular RNA at 24 hpi, and determined RNase L activation by cleavage of ribosomal RNA (rRNA), as indicated by the Agilent RNA Nano 6000 assay (Figure 5a). RNase L was weakly activated during infection with BF785 compared to wtMERS, as shown by the slight 28S and 18S rRNA degradation. Given the severe attenuation of the Mor213 virus, the lack of RNase L activation measured by this assay could be confounded by low viral replication. Thus, to probe RNase L activation at the individual cell level, we next utilized a correlate of RNase L activation by assessing nuclear translocation of poly(A)-binding protein cytoplasmic 1 (PABPC1) as a more sensitive assay. PABPC1 is a highly conserved RNA binding protein that binds to the 3′ poly(A) tail of host mRNAs and has been shown to translocate to the nucleus upon RNase L activation, thereby serving as a correlate of induction [54]. We infected A549^DPP4^ cells with all four viruses (MOI = 0.5 PFU/cell, n = 3) and fixed the cells at 24 hpi (Figure 5b).

Subsequently, we stained for MERS-CoV N (green), PABPC1 (red), and DAPI (blue) and quantified percent of infected cells (single cells and syncytia) with nuclear PABPC1. Infected cells positive for RNase L activation have nuclei that appear pink/purple due to the overlap between DAPI and PABPC1 (red). We also infected A549^DPP4^ cells with knockout (KO) of the *RNASEL* gene in parallel with all four viruses to confirm the PABPC1 translocation observed was RNase L-dependent (MOI = 0.5 PFU/cell, n = 3) (Supplementary Figure S3).

wtMERS-infected cells exhibited the most infection and, as expected, little-to-no nuclear PABPC1. As in the titer data, Mor213 showed the least amount of infection and intermediate levels of nuclear PABPC1 consistent with the deletions in the PDE domain. BF785 shows the greatest amount of nuclear PABPC1, comparable to MERS-ΔNS4b. Interestingly, we also found that BF785 induces more of a cytopathic effect (CPE) and more syncytium formation than the other three viruses.

### 3.5. Knockout of RNase L, but Not PKR or MAVS, Restores the Replication Deficit for BF785, but Not Mor213

Due to replication defects observed in A549^DPP4^ and Calu-3 cells and the strong innate immunostimulatory phenotype observed during infection with BF785, we sought to determine whether knockout (KO) of a key mediator in each of the three double-stranded RNA-induced antiviral pathways involved in HCoV sensing—OAS-RNase L, PKR, and MDA5—could rescue replication of the West African camel strains. PKR, a serine/threonine kinase constitutively expressed in all tissues, dimerizes and autophosphorylates upon detecting dsRNA before phosphorylating translation initiation factor eIF2a (p-eIF2a), leading to protein synthesis attenuation and restriction of viral replication [55]. In wtMERS-CoV infection, the PKR pathway is not activated [38,46,56]. MERS NS4a, along with the coronavirus-conserved endoribonuclease (EndoU) in nonstructural protein nsp15, promotes the shutdown of dsRNA-induced pathways by binding to and shielding viral dsRNA from detection [37,38]. NS4a is thought to protect dsRNA from detection and, along with nsp15, dampens IFN signaling, as well as activation of the OAS-RNase L and PKR pathways [57].

**Figure 6:**
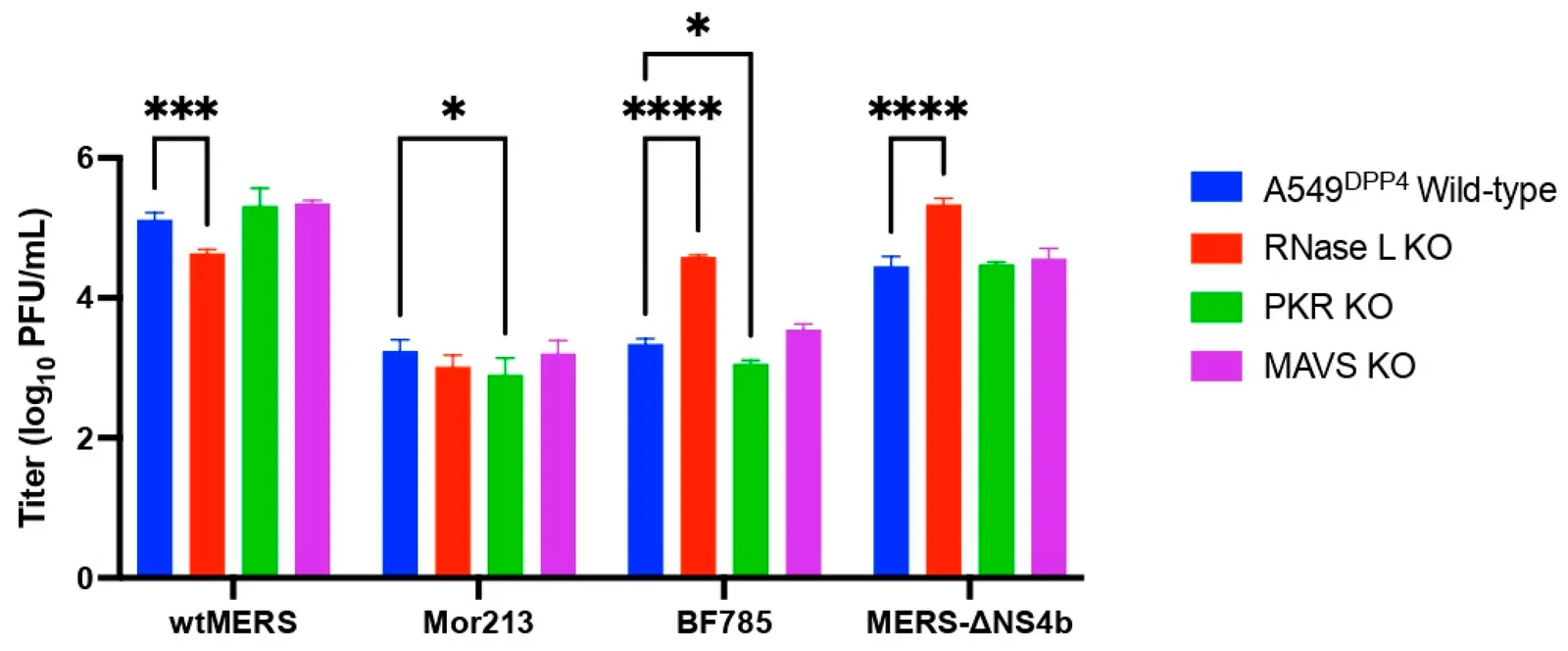
A549^DPP4^ cell lines: Wild-type, RNase L KO, PKR KO, and MAVS KO were infected (MOI = 0.1 PFU/cell, n = 3) with wtMERS, Mor213, BF785, and MERS-ΔNS4b, as well as supernatant samples collected at 24 hpi. Viral replication was quantified by plaque assay. Data are displayed as means ± SD. Statistical significance of differences in viral replication for Mor213, BF785, and MERS-ΔNS4b compared to wtMERS was calculated by repeated-measures two-way ANOVA: * *p* ≤ 0.05, *** *p* = 0.0003, and **** *p* ≤ 0.0001. Data that were not statistically significant are not labeled. (n = 3. Data are from three independent experiments combined).

We previously constructed A549^DPP4^ RNase L KO cells, A549^DPP4^ PKR KO cells, and A549^DPP4^ MAVS KO cells to interrogate the host RNase L, PKR, and MDA5 pathways, respectively [37,38]. We compared replication of wtMERS, Mor213, BF785, and MERS-ΔNS4b in all four cell lines (Figure 6). Cells were infected at an MOI of 0.1 PFU/cell, and supernatant samples were harvested at 24 hpi. Quantification of infectious virus was performed by plaque assay.

Knockout of PKR in A549^DPP4^ did not rescue Mor213, BF785, or MERS-ΔNS4b replication, consistent with the antagonism of the PKR pathways by NS4a, which is retained in these viruses. Similarly, knocking out MAVS did not rescue Mor213, BF785, or MERS-ΔNS4b replication. Knockout of *RNASEL* almost completely rescued replication of BF785, consistent with its stronger activation of RNase L that we observed in the CHIP and the PABPC1 assay. Interestingly, knockout of *RNASEL* completely rescued replication of MERS-ΔNS4b, despite its reduced activation of RNase L relative to BF785. Knockout of *RNASEL* did not rescue Mor213 replication.

## 4. Discussion

MERS-CoV clade C viruses circulate widely in dromedary camels across Africa but have not been associated with human disease or sustained human-to-human transmission. This contrasts with the clade A/B viruses, the latter of which continue to cause outbreaks in the Middle East. In this study, we demonstrate that two West African clade C isolates with distinct deletions in *ORF4b*, Mor213 and BF785, exhibit attenuated replication in human respiratory epithelial cell lines. We also show that these viruses are attenuated in human primary bronchial ALI cultures, albeit in a donor-dependent manner. Moreover, infections with Mor213 and BF785 in both cell lines and bronchial ALI cultures were accompanied by varying degrees of innate immune activation. Finally, we show that the BF785 strain, which harbors a complete *ORF4b* deletion, induces significant activation of the antiviral protein RNase L, and that *RNASEL* knockout rescues replication of BF785 comparably to that of wtMERS. A limitation of this study is that we did not include a clade B virus as an additional comparator. Currently, zoonotic disease in the Arabian Peninsula is primarily caused by clade B viruses, which replicate similarly to clade A wtMERS in human cells.

*ORF4b* encodes NS4b, which antagonizes IFN signaling through two mechanisms: NLS-mediated blockade of NFKB translocation and PDE-driven degradation of 2-5A, preventing activation of RNase L [35,37,38,39,42,43,44]. Consistent with the absence of the NLS, BF785 induces more robust IFN signaling and inflammatory marker mRNA expression than both wtMERS and Mor213 in Calu-3 cells, comparable to the MERS-ΔNS4b mutant (Figure 3). At the protein level, Mor213, which contains a partial deletion in *ORF4b* but retains the NLS sequence, appears to induce similar levels of IFN and ISG expression (Figure 3c).

In primary human bronchial ALI cultures, the West African clade C isolates exhibit attenuated replication compared to wtMERS in three of five donors, consistent with previous findings using ex vivo bronchial cultures (Figure 4a) [11]. However, in two of five donors (8000 and 8015), all three viruses (wtMERS, Mor213, and BF785) exhibited low and comparable replication, suggesting there are donor-dependent differences in susceptibility to MERS-CoV, consistent with previous findings in our lab and others [46,58,59]. It has been reported that differences in DPP4 expression in the human airway correlate with MERS severity [58]. Separately, the level of soluble DPP4 (sDPP4) was found to be reduced in the serum of MERS-CoV-infected patients, while high levels of sDPP4 were suggested to be protective against MERS-CoV in an animal model of infection [58,60]. Whether donor-to-donor differences in DPP4 expression or additional factors contribute to the donor–donor variability of MERS-CoV susceptibility remains to be examined. Interestingly, we observed that the cultures from donors that exhibited the least MERS-CoV replication also exhibited lower levels of IFN and ISG induction at the protein level (Figure 4b). We also observed some induction of ISGs with wtMERS in bronchial ALI cultures in certain donors, although less than the clade C viruses. Previously, we observed very little ISG induction with wtMERS in primary nasal epithelial cultures, suggesting there are differences in ISG responses between the upper and lower airway [46]. In the three bronchial donors where the West African viruses were attenuated relative to wtMERS, we observed a trend towards increased IFN induction relative to wtMERS-infected cultures (Figure 4b). This suggests that both the partial and full deletion of *ORF4b* results in an increase in IFN induction, which likely contributes to the attenuation of these viruses in the human airway. While Mor213 retains the NLS of NS4b, there is a single amino acid substitution, V35F. This substitution retains its hydrophobicity; however, its effects, if any, are unknown. We are actively investigating differences in ISG induction upon wtMERS infection in the upper and lower airway, in addition to donor-dependent differences in susceptibility to MERS-CoV.

Furthermore, consistent with the absence of the NS4b PDE, we show that BF785 infection results in activation of the OAS-RNase L pathway (Figure 5). Mor213 also displays moderate activation of RNase L, in agreement with the deletions sustained in the PDE domain—however, to a lesser extent than BF785. When we knockout each of three dsRNA-sensing innate immune pathways (RNase L, PKR, and MAVS) in A549^DPP4^ cells, BF785 replication is rescued by *RNASEL* knockout, while Mor213 replication remains attenuated across the cell lines. We have previously reported that Mor213 is particularly attenuated in human cell lines and even shows attenuation in Vero-CCL81 cells [30]. We hypothesize that there are additional constraints on Mor213 replication beyond innate sensing and IFN antagonism, and that the particularly low replication of Mor213 may explain the reduced RNase L activation observed with this virus, despite the absence of a PDE domain in NS4b. We previously reported that clade A wtMERS nearly completely suppresses the dsRNA-induced IFN and signaling pathways, PKR, and RNase L through the combined activities of nsp15 EndoU and NS4a and NS4b in cell lines and in primary nasal epithelial cultures [38,56]. Mutation of one or more of the genes encoding these antagonists leads to activation of antiviral pathways and attenuation of replication. PKR and MAVS KO likely do not rescue Mor213 and BF785 replication because both viruses retain nsp15 EndoU and NS4a.

Apart from *ORF4b*, Mor213 and BF785 also encode amino acid substitutions in other accessory and/or nonstructural proteins which have been implicated in antagonizing the interferon response. Among the accessory proteins, the exact function of NS3 is still not entirely understood; meanwhile, NS5 has been suggested to function as a viroporin and alter NFκB-mediated inflammation [44,45]. Both Mor213 and BF785 contain substitutions in the genes encoding these proteins, which may also contribute to the increased ISG responses induced by these viruses and requires further investigation. Importantly, no substitutions occur in nsp15 catalytic residues or in nsp6 in our panel of viruses—amino acid substitution L232F in nsp6 is potentially involved in MERS-CoV human adaptation [61]. Additionally, these viruses contain several synonymous and non-synonymous mutations in other nonstructural proteins, which may also contribute to their poor replication in humans. The strong induction of ISGs by BF785 shown here further underlines the importance of these accessory genes in antagonizing the IFN response.

The mechanism by which these deletions in key accessory genes are selected for in camel populations and whether variants containing these deletions continue to circulate in camels warrant further investigation. Camelids have been found to lack OAS2 and OAS3, but they retain OAS1, OASL, OAS4, and RNase L [62]. Whether the lack of OAS2 and OAS3 has any functional consequence on MERS-CoV evolution in camels is currently unknown. While *ORF4b* deletions are rare in clade A/B MERS-CoV strains circulating in the Middle East, multiple sequences with premature stop codons in *ORF4b* have been reported in 2012, 2015, and 2018, suggesting there are specific scenarios where deletion of *ORF4b* can arise and perhaps even be advantageous [63,64]. Although there are significantly more MERS-CoV sequences available from the Middle East compared to West Africa, the relatively high proportion of *ORF4b* deletions in West Africa compared to the Middle East could suggest different constraints on viral transmission and persistence in camel populations between the Middle East and Africa. It has been previously suggested that differences in camel herding and slaughterhouse practices contribute to the decreased MERS-CoV transmissibility in East Africa relative to Saudi Arabia [15]. Differences in camel herding and farming, which would alter population density, could exert different bottlenecks on virus transmission and evolution. Understanding the transmission potential of clade C viruses containing *ORF4b* deletions in camels and what drives the persistence of these viruses in camel herds remains an important question in understanding the pandemic potential of clade C MERS-CoV strains.

Altogether, these findings provide mechanistic insight into the reduced fitness of West African clade C MERS-CoV in human cells and highlight functional differences between natural NS4b variants. We show that naturally occurring deletions in *ORF4b* of clade C MERS-CoV strains result in increased induction of ISGs and activation of RNase L in lung-derived cell lines and, primary, bronchial epithelial cells in a donor-dependent manner. We suggest that the defects in evading antiviral sensing shown here may contribute to the absence of reported human outbreaks caused by clade C viruses in West Africa. Understanding these virus–host interactions will continue to shed light on the evolution of MERS-CoV strains between host species.

## Data Availability

The original contributions presented in this study are included in the article/Supplementary Materials. Further inquiries can be directed to the corresponding authors. RNAseq data can be located with the GEO accession number: GSE341609.

## Supplementary Materials

The following supporting information can be downloaded at https://www.mdpi.com/article/10.3390/v18090935/s1. Supplementary Figure S1. Individual donor bronchial ALI cultures were infected at the apical surface in triplicate with the indicated viruses (MOI = 0.1 PFU/cell, 37 °C). Whole cell lysates were collected at 96 hpi and analyzed by western blot. Relative target protein expression normalized to GAPDH was quantified in ImageJ Version 1.54p by donor and plotted. Supplementary Figure S2. A549^DPP4^ cells were infected in duplicate at MOI = 0.5 PFU/cell with wtMERS, Mor213, BF785, and MERS-ΔNS4b, fixed 24 hpi in 4% PFA, and stained for MERS N (green), PABPC1 (red) and DAPI (blue). Images were acquired on a Nikon Eclipse Ti 2. All experiments are n = 3 in technical duplicate with 6 images taken per condition per experiment. Supplementary Figure S3. A549^DPP4^ *RNASEL* KO cells were infected in duplicate at MOI = 0.5 PFU/cell with wtMERS, Mor213, BF785, and MERS-ΔNS4b, fixed 24 hpi in 4% PFA, and stained for MERS N (green), PABPC1 (red) and DAPI (blue). Images were acquired on a Nikon Eclipse Ti 2. All experiments are n = 3 in technical duplicate with 6 images taken per condition per experiment.

## Abbreviations

The following abbreviations are used in this manuscript:

MERS-CoV	Middle East respiratory syndrome coronavirus
OAS-RNase L	Oligoadenylate synthetase–ribonuclease L
WHO	World Health Organization
EMC	Erasmus Medical Center
HCoV	Human coronavirus
PDE	Phosphodiesterase
NLS	Nuclear localization sequence
DMEM	Dulbecco’s Modified Eagle Medium
FBS	Fetal bovine serum
MOI	Multiplicity of infection
PFU	Plaque-forming units
PBS	Phosphate-buffered saline
hpi	Hours post-infection
ASL	Apical surface liquid
ALI	Air–liquid interface
IFN	Interferon
ISG	Interferon-stimulated gene
SDS/PAGE	Sodium dodecyl sulfate–polyacrylamide gel electrophoresis
PVDF	Polyvinylidene difluoride
BSA	Bovine serum albumin
PABPC1	Poly(A)-Binding Protein Cytoplasmic 1
KO	Knockout

## Appendix A

**Table A1:** Primers used for qPCR analysis.

Gene Name	Primer Orientation	Sequence
IFNL1	Forward	CGCCTTGGAAGAGTCACTCA
IFNL1	Reverse	GAAGCCTCAGGTCCCAATTC
IFNB	Forward	GTCAGAGTGGAAATCCTAAG
IFNB	Reverse	ACAGCATCTGCTGGTTGAAG
IFIT1	Forward	TGGTGACCTGGGGCAACTTT
IFIT1	Reverse	AGGCCTTGGCCCGTTCATAA
RSAD2	Forward	CACAAAGAAGTGTCCTGCTTGGT
RSAD2	Reverse	AAGCGCATATATTCATCCAGAATAAG
CXCL10	Forward	CCTGCAAGCCAATTTTGTCC
CXCL10	Reverse	ATGGCCTTCGATTCTGGATTC
TNFα	Forward	CCTCTCTCTAATCAGCCCTCTG
TNFα	Reverse	GAGGACCTGGGAGTAGATGAG
IL-6	Forward	GCCCAGCTATGAACTCCTTCT
IL-6	Reverse	GCGGCTACATCTTTGGAATCT
18S	Forward	TTCGATGGTAGTCGCTGTGC
18S	Reverse	CTGCTGCCTTCCTTGAATGTGGTA

**Table A2:** Primary antibodies used for Western blots and immunofluorescence imaging.

Primary Antibody	Species	Blocking Buffer	Dilution	Cat. No.
MDA5	Rabbit	5% milk in TBST	1:1000	Cell Signaling 5321
p-STAT1	Rabbit	5% BSA	1:1000	Cell Signaling 7649
STAT1	Rabbit	5% BSA	1:1000	Cell Signaling 9172
p-PKR	Rabbit	5% BSA	1:1000	Abcam 32036
PKR	Rabbit	5% BSA	1:1000	Cell Signaling 12297
Viperin	Rabbit	5% milk in TBST	1:1000	Cell Signaling 13996
IFIT1	Rabbit	5% milk in TBST	1:1000	Cell Signaling 14769
MERS N	Mouse	5% milk in TBST	1:2000	Sino Biological 40068-MM10
GAPDH	Rabbit	5% milk in TBST	1:2000	Cell Signaling 2118
PABPC1	Rabbit	5% milk in TBST	1:1000	Abcam

**Table A3:** Secondary antibodies used for Western blots and immunofluorescence imaging.

Secondary Antibody	Species	Blocking Buffer	Dilution	Cat. No.
Anti-rabbit IgG HRP-linked	Goat	Same as primary	1:3000	Cell Signaling 7074
Anti-mouse IgG HRP-linked	Horse	Same as primary	1:3000	Cell Signaling 7076

## References

[1] Zumla A., Hui D.S., Peiris M., Perlman S. (2026). Respiratory infections due to human common cold coronaviruses, SARS-CoV, MERS-CoV, and SARS-CoV-2: Epidemiology, pathogenesis, clinical features, diagnostics, therapeutics, and vaccine landscapes. Lancet Respir. Med..

[2] Perera R.A., Wang P., Gomaa M.R., El-Shesheny R., Kandeil A., Bagato O., Siu L.Y., Shehata M.M., Kayed A.S., Moatasim Y. (2013). Seroepidemiology for MERS coronavirus using microneutralisation and pseudoparticle virus neutralisation assays reveal a high prevalence of antibody in dromedary camels in Egypt, June 2013. Eurosurveillance.

[3] Assiri A., McGeer A., Perl T.M., Price C.S., Al Rabeeah A.A., Cummings D.A., Alabdullatif Z.N., Assad M., Almulhim A., Makhdoom H. (2013). Hospital outbreak of Middle East respiratory syndrome coronavirus. N. Engl. J. Med..

[4] Azhar E.I., El-Kafrawy S.A., Farraj S.A., Hassan A.M., Al-Saeed M.S., Hashem A.M., Madani T.A. (2014). Evidence for camel-to-human transmission of MERS coronavirus. N. Engl. J. Med..

[5] Azhar E.I., Velavan T.P., Rungsung I., Traore T., Hui D.S., McCloskey B., El-Kafrawy S.A., Zumla A. (2023). Middle East respiratory syndrome coronavirus-a 10-year (2012–2022) global analysis of human and camel infections, genomic sequences, lineages, and geographical origins. Int. J. Infect. Dis..

[6] Haagmans B.L., Al Dhahiry S.H., Reusken C.B., Raj V.S., Galiano M., Myers R., Godeke G.J., Jonges M., Farag E., Diab A. (2014). Middle East respiratory syndrome coronavirus in dromedary camels: An outbreak investigation. Lancet Infect. Dis..

[7] WHO Middle East Respiratory Syndrome Coronavirus (MERS-CoV). https://www.who.int/news-room/fact-sheets/detail/middle-east-respiratory-syndrome-coronavirus-(mers-cov).

[8] Chu D.K.W., Hui K.P.Y., Perera R., Miguel E., Niemeyer D., Zhao J., Channappanavar R., Dudas G., Oladipo J.O., Traore A. (2018). MERS coronaviruses from camels in Africa exhibit region-dependent genetic diversity. Proc. Natl. Acad. Sci. USA.

[9] Te N., Rodon J., Perez M., Segales J., Vergara-Alert J., Bensaid A. (2022). Enhanced replication fitness of MERS-CoV clade B over clade A strains in camelids explains the dominance of clade B strains in the Arabian Peninsula. Emerg. Microbes Infect..

[10] So R.T.Y., Hui K.P.Y., Ho J.C.W., Lau K.K.M., Zhou Z., Chan M.C.W., Poon L.L.M., Peiris M. (2026). The impact of clade B lineage 5 MERS coronaviruses spike mutations from 2015 to 2023 on virus entry and replication competence. PLoS Pathog..

[11] Zhou Z., Hui K.P.Y., So R.T.Y., Lv H., Perera R., Chu D.K.W., Gelaye E., Oyas H., Njagi O., Abayneh T. (2021). Phenotypic and genetic characterization of MERS coronaviruses from Africa to understand their zoonotic potential. Proc. Natl. Acad. Sci. USA.

[12] Zhou Z., Ali A., Walelign E., Demissie G.F., El Masry I., Abayneh T., Getachew B., Krishnan P., Ng D.Y.M., Gardner E. (2023). Genetic diversity and molecular epidemiology of Middle East Respiratory Syndrome Coronavirus in dromedaries in Ethiopia, 2017–2020. Emerg. Microbes Infect..

[13] Tanneti N.S., Stillwell H.A., Weiss S.R. (2025). Human coronaviruses: Activation and antagonism of innate immune responses. Microbiol. Mol. Biol. Rev..

[14] Hemida M.G., Al-Naeem A., Perera R.A., Chin A.W., Poon L.L., Peiris M. (2015). Lack of middle East respiratory syndrome coronavirus transmission from infected camels. Emerg. Infect. Dis..

[15] Ogoti B., Riitho V., Wildemann J., Mutono N., Mureithi M., Oyugi J., Rodon J., Corman V.M., Drosten C., Thumbi S.M. (2026). Epidemiology and genomic features of MERS coronavirus in Africa: A systematic and meta-analysis review. Int. J. Infect. Dis..

[16] Peiris M., Perlman S. (2022). Unresolved questions in the zoonotic transmission of MERS. Curr. Opin. Virol..

[17] Munyua P., Corman V.M., Bitek A., Osoro E., Meyer B., Muller M.A., Lattwein E., Thumbi S.M., Murithi R., Widdowson M.A. (2017). No Serologic Evidence of Middle East Respiratory Syndrome Coronavirus Infection Among Camel Farmers Exposed to Highly Seropositive Camel Herds: A Household Linked Study, Kenya, 2013. Am. J. Trop. Med. Hyg..

[18] Liljander A., Meyer B., Jores J., Muller M.A., Lattwein E., Njeru I., Bett B., Drosten C., Corman V.M. (2016). MERS-CoV Antibodies in Humans, Africa, 2013–2014. Emerg. Infect. Dis..

[19] So R.T., Perera R.A., Oladipo J.O., Chu D.K., Kuranga S.A., Chan K.H., Lau E.H., Cheng S.M., Poon L.L., Webby R.J. (2018). Lack of serological evidence of Middle East respiratory syndrome coronavirus infection in virus exposed camel abattoir workers in Nigeria, 2016. Eurosurveill.

[20] Ogoti B.M., Riitho V., Wildemann J., Mutono N., Tesch J., Rodon J., Harichandran K., Emanuel J., Moncke-Buchner E., Kiambi S. (2024). Biphasic MERS-CoV Incidence in Nomadic Dromedaries with Putative Transmission to Humans, Kenya, 2022–2023. Emerg. Infect. Dis..

[21] Karani A., Ombok C., Situma S., Breiman R., Mureithi M., Jaoko W., Njenga M.K., Ngere I. (2025). Low-Level Zoonotic Transmission of Clade C MERS-CoV in Africa: Insights from Scoping Review and Cohort Studies in Hospital and Community Settings. Viruses.

[22] Karani A., Ngere I., Ombok C., Singh D., Jaoko W., Njenga M.K., Palmer G.H. (2025). Low Prevalence of Middle East Respiratory Syndrome Coronavirus Infection in Camel-Exposed Patients Presenting with Respiratory Symptoms in Northern Kenya. Am. J. Trop. Med. Hyg..

[23] Chu D.K., Poon L.L., Gomaa M.M., Shehata M.M., Perera R.A., Abu Zeid D., El Rifay A.S., Siu L.Y., Guan Y., Webby R.J. (2014). MERS coronaviruses in dromedary camels, Egypt. Emerg. Infect. Dis..

[24] Abbad A., Perera R.A., Anga L., Faouzi A., Minh N.N.T., Malik S., Iounes N., Maaroufi A., Van Kerkhove M.D., Peiris M. (2019). Middle East respiratory syndrome coronavirus (MERS-CoV) neutralising antibodies in a high-risk human population, Morocco, November 2017 to January 2018. Eurosurveill.

[25] Mok C.K.P., Zhu A., Zhao J., Lau E.H.Y., Wang J., Chen Z., Zhuang Z., Wang Y., Alshukairi A.N., Baharoon S.A. (2021). T-cell responses to MERS coronavirus infection in people with occupational exposure to dromedary camels in Nigeria: An observational cohort study. Lancet Infect. Dis..

[26] Munyua P.M., Ngere I., Hunsperger E., Kochi A., Amoth P., Mwasi L., Tong S., Mwatondo A., Thornburg N., Widdowson M.A. (2021). Low-Level Middle East Respiratory Syndrome Coronavirus among Camel Handlers, Kenya, 2019. Emerg. Infect. Dis..

[27] Ngere I., Hunsperger E.A., Tong S., Oyugi J., Jaoko W., Harcourt J.L., Thornburg N.J., Oyas H., Muturi M., Osoro E.M. (2022). Outbreak of Middle East Respiratory Syndrome Coronavirus in Camels and Probable Spillover Infection to Humans in Kenya. Viruses.

[28] Miguel E., Perera R.A., Baubekova A., Chevalier V., Faye B., Akhmetsadykov N., Ng C.Y., Roger F., Peiris M. (2016). Absence of Middle East Respiratory Syndrome Coronavirus in Camelids, Kazakhstan, 2015. Emerg. Infect. Dis..

[29] Chan S.M., Damdinjav B., Perera R.A., Chu D.K., Khishgee B., Enkhbold B., Poon L.L., Peiris M. (2015). Absence of MERS-Coronavirus in Bactrian Camels, Southern Mongolia, November 2014. Emerg. Infect. Dis..

[30] Winstone H., Stillwell H., Tan L.H., Cohen N.A., Perera R., Weiss S.R. (2026). Clade C MERS-CoV camel strains vary in protease utilization during viral entry. Proc. Natl. Acad. Sci. USA.

[31] Brian D.A., Baric R.S. (2005). Coronavirus genome structure and replication. Curr. Top. Microbiol. Immunol..

[32] Cotten M., Lam T.T., Watson S.J., Palser A.L., Petrova V., Grant P., Pybus O.G., Rambaut A., Guan Y., Pillay D. (2013). Full-genome deep sequencing and phylogenetic analysis of novel human betacoronavirus. Emerg. Infect. Dis..

[33] Roth-Cross J.K., Stokes H., Chang G., Chua M.M., Thiel V., Weiss S.R., Gorbalenya A.E., Siddell S.G. (2009). Organ-specific attenuation of murine hepatitis virus strain A59 by replacement of catalytic residues in the putative viral cyclic phosphodiesterase ns2. J. Virol..

[34] Weiss S.R., Navas-Martin S. (2005). Coronavirus pathogenesis and the emerging pathogen severe acute respiratory syndrome coronavirus. Microbiol. Mol. Biol. Rev..

[35] Thornbrough J.M., Jha B.K., Yount B., Goldstein S.A., Li Y., Elliott R., Sims A.C., Baric R.S., Silverman R.H., Weiss S.R. (2016). Middle East Respiratory Syndrome Coronavirus NS4b Protein Inhibits Host RNase L Activation. mBio.

[36] Zhao L., Jha B.K., Wu A., Elliott R., Ziebuhr J., Gorbalenya A.E., Silverman R.H., Weiss S.R. (2012). Antagonism of the interferon-induced OAS-RNase L pathway by murine coronavirus ns2 protein is required for virus replication and liver pathology. Cell Host Microbe.

[37] Comar C.E., Goldstein S.A., Li Y., Yount B., Baric R.S., Weiss S.R. (2019). Antagonism of dsRNA-Induced Innate Immune Pathways by NS4a and NS4b Accessory Proteins during MERS Coronavirus Infection. mBio.

[38] Comar C.E., Otter C.J., Pfannenstiel J., Doerger E., Renner D.M., Tan L.H., Perlman S., Cohen N.A., Fehr A.R., Weiss S.R. (2022). MERS-CoV endoribonuclease and accessory proteins jointly evade host innate immunity during infection of lung and nasal epithelial cells. Proc. Natl. Acad. Sci. USA.

[39] Goldstein S.A., Thornbrough J.M., Zhang R., Jha B.K., Li Y., Elliott R., Quiroz-Figueroa K., Chen A.I., Silverman R.H., Weiss S.R. (2017). Lineage A Betacoronavirus NS2 Proteins and the Homologous Torovirus Berne pp1a Carboxy-Terminal Domain Are Phosphodiesterases That Antagonize Activation of RNase L. J. Virol..

[40] Li Y., Banerjee S., Goldstein S.A., Dong B., Gaughan C., Rath S., Donovan J., Korennykh A., Silverman R.H., Weiss S.R. (2017). Ribonuclease L mediates the cell-lethal phenotype of double-stranded RNA editing enzyme ADAR1 deficiency in a human cell line. eLife.

[41] Silverman R.H. (2007). Viral encounters with 2′,5′-oligoadenylate synthetase and RNase L during the interferon antiviral response. J. Virol..

[42] Canton J., Fehr A.R., Fernandez-Delgado R., Gutierrez-Alvarez F.J., Sanchez-Aparicio M.T., Garcia-Sastre A., Perlman S., Enjuanes L., Sola I. (2018). MERS-CoV 4b protein interferes with the NF-kappaB-dependent innate immune response during infection. PLoS Pathog..

[43] Matthews K.L., Coleman C.M., van der Meer Y., Snijder E.J., Frieman M.B. (2014). The ORF4b-encoded accessory proteins of Middle East respiratory syndrome coronavirus and two related bat coronaviruses localize to the nucleus and inhibit innate immune signalling. J. Gen. Virol..

[44] Yang Y., Zhang L., Geng H., Deng Y., Huang B., Guo Y., Zhao Z., Tan W. (2013). The structural and accessory proteins M, ORF 4a, ORF 4b, and ORF 5 of Middle East respiratory syndrome coronavirus (MERS-CoV) are potent interferon antagonists. Protein Cell.

[45] Menachery V.D., Mitchell H.D., Cockrell A.S., Gralinski L.E., Yount B.L., Graham R.L., McAnarney E.T., Douglas M.G., Scobey T., Beall A. (2017). MERS-CoV Accessory ORFs Play Key Role for Infection and Pathogenesis. mBio.

[46] Otter C.J., Renner D.M., Fausto A., Tan L.H., Cohen N.A., Weiss S.R. (2024). Interferon signaling in the nasal epithelium distinguishes among lethal and common cold coronaviruses and mediates viral clearance. Proc. Natl. Acad. Sci. USA.

[47] Scobey T., Yount B.L., Sims A.C., Donaldson E.F., Agnihothram S.S., Menachery V.D., Graham R.L., Swanstrom J., Bove P.F., Kim J.D. (2013). Reverse genetics with a full-length infectious cDNA of the Middle East respiratory syndrome coronavirus. Proc. Natl. Acad. Sci. USA.

[48] Liberzon A., Birger C., Thorvaldsdottir H., Ghandi M., Mesirov J.P., Tamayo P. (2015). The Molecular Signatures Database (MSigDB) hallmark gene set collection. Cell Syst..

[49] Zielecki F., Weber M., Eickmann M., Spiegelberg L., Zaki A.M., Matrosovich M., Becker S., Weber F. (2013). Human cell tropism and innate immune system interactions of human respiratory coronavirus EMC compared to those of severe acute respiratory syndrome coronavirus. J. Virol..

[50] Chan R.W., Chan M.C., Agnihothram S., Chan L.L., Kuok D.I., Fong J.H., Guan Y., Poon L.L., Baric R.S., Nicholls J.M. (2013). Tropism of and innate immune responses to the novel human betacoronavirus lineage C virus in human ex vivo respiratory organ cultures. J. Virol..

[51] Kindler E., Jonsdottir H.R., Muth D., Hamming O.J., Hartmann R., Rodriguez R., Geffers R., Fouchier R.A., Drosten C., Muller M.A. (2013). Efficient replication of the novel human betacoronavirus EMC on primary human epithelium highlights its zoonotic potential. mBio.

[52] Adivitiya, Kaushik M.S., Chakraborty S., Veleri S., Kateriya S. (2021). Mucociliary Respiratory Epithelium Integrity in Molecular Defense and Susceptibility to Pulmonary Viral Infections. Biology.

[53] Otter C.J., Fausto A., Tan L.H., Khosla A.S., Cohen N.A., Weiss S.R. (2023). Infection of primary nasal epithelial cells differentiates among lethal and seasonal human coronaviruses. Proc. Natl. Acad. Sci. USA.

[54] Burke J.M., Moon S.L., Matheny T., Parker R. (2019). RNase L Reprograms Translation by Widespread mRNA Turnover Escaped by Antiviral mRNAs. Mol. Cell.

[55] Hur S. (2019). Double-Stranded RNA Sensors and Modulators in Innate Immunity. Annu. Rev. Immunol..

[56] Otter C.J., Bracci N., Parenti N.A., Ye C., Asthana A., Blomqvist E.K., Tan L.H., Pfannenstiel J.J., Jackson N., Fehr A.R. (2024). SARS-CoV-2 nsp15 endoribonuclease antagonizes dsRNA-induced antiviral signaling. Proc. Natl. Acad. Sci. USA.

[57] Rabouw H.H., Langereis M.A., Knaap R.C., Dalebout T.J., Canton J., Sola I., Enjuanes L., Bredenbeek P.J., Kikkert M., de Groot R.J. (2016). Middle East Respiratory Coronavirus Accessory Protein 4a Inhibits PKR-Mediated Antiviral Stress Responses. PLoS Pathog..

[58] Breugem T.I., Riesebosch S., Zhang J., Mykytyn A.Z., Krabbendam L., Groen N., Baptista Varela S., Schipper D., van den Doel P.B., van Acker R. (2025). Variable DPP4 expression in multiciliated cells of the human nasal epithelium as a determinant for MERS-CoV tropism. Proc. Natl. Acad. Sci. USA.

[59] Li K., Wohlford-Lenane C., Bartlett J.A., McCray P.B. (2021). Inter-individual Variation in Receptor Expression Influences MERS-CoV Infection and Immune Responses in Airway Epithelia. Front. Public Health.

[60] Alkharsah K.R., Aljaroodi S.A., Rahman J.U., Alnafie A.N., Al Dossary R., Aljindan R.Y., Alnimr A.M., Hussen J. (2022). Low levels of soluble DPP4 among Saudis may have constituted a risk factor for MERS endemicity. PLoS ONE.

[61] So R.T.Y., Chu D.K.W., Hui K.P.Y., Mok C.K.P., Shum M.H.H., Sanyal S., Nicholls J.M., Ho J.C.W., Cheung M.C., Ng K.C. (2023). Amino acid substitution L232F in non-structural protein 6 identified as a possible human-adaptive mutation in clade B MERS coronaviruses. J. Virol..

[62] Eckhart L., Sipos W. (2023). Differential Loss of OAS Genes Indicates Diversification of Antiviral Immunity in Mammals. Vaccines.

[63] El-Kafrawy S.A., Corman V.M., Tolah A.M., Al Masaudi S.B., Hassan A.M., Muller M.A., Bleicker T., Harakeh S.M., Alzahrani A.A., Alsaaidi G.A. (2019). Enzootic patterns of Middle East respiratory syndrome coronavirus in imported African and local Arabian dromedary camels: A prospective genomic study. Lancet Planet. Health.

[64] Ba Abduallah M.M., Hemida M.G. (2021). Comparative analysis of the genome structure and organization of the Middle East respiratory syndrome coronavirus (MERS-CoV) 2012 to 2019 revealing evidence for virus strain barcoding, zoonotic transmission, and selection pressure. Rev. Med. Virol..

