# Supplementary material for: West African Clade C MERS-CoV Strains BF785 and Mor213 Induce More Robust Interferon and Inflammatory Signaling Compared to Clade A in Human Respiratory Cells
Source: Viruses. 2026 Aug 27;18(9):935. doi: 10.3390/v18090935 (PMC13612015; doi:10.3390/v18090935)
Supplement: Supplementary file 1 [file viruses-18-00935-s001.zip › viruses-4494963-supplementary.pdf]

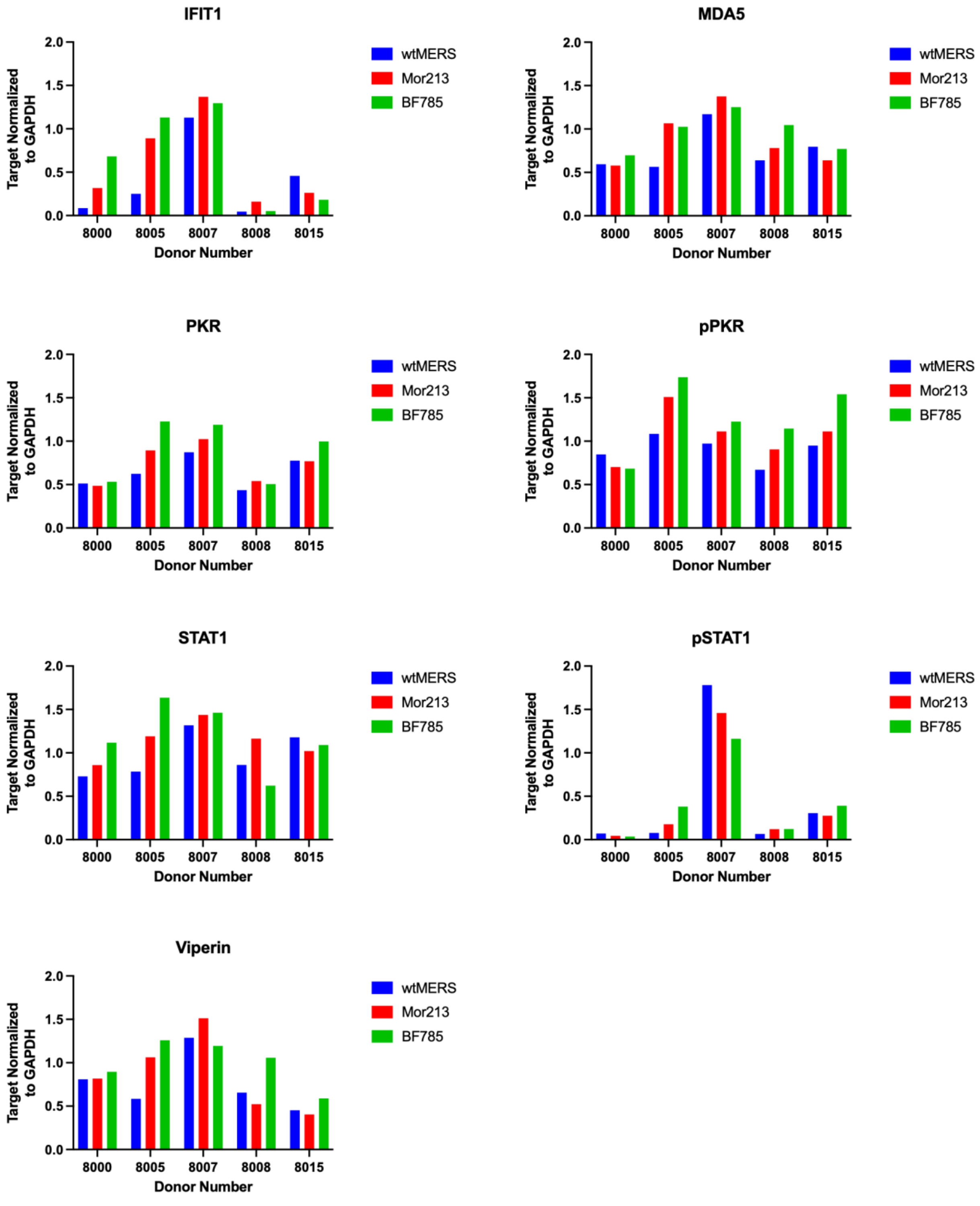

Supplementary Figure S1. Individual donor bronchial ALI cultures were infected at the apical surface in triplicate with the indicated viruses (MOI = 0.1 PFU/cell, 37°C). Whole cell lysates were collected at 96 hpi and analyzed by western blot. Relative target protein expression normalized to GAPDH was quantified in ImageJ by donor and plotted.

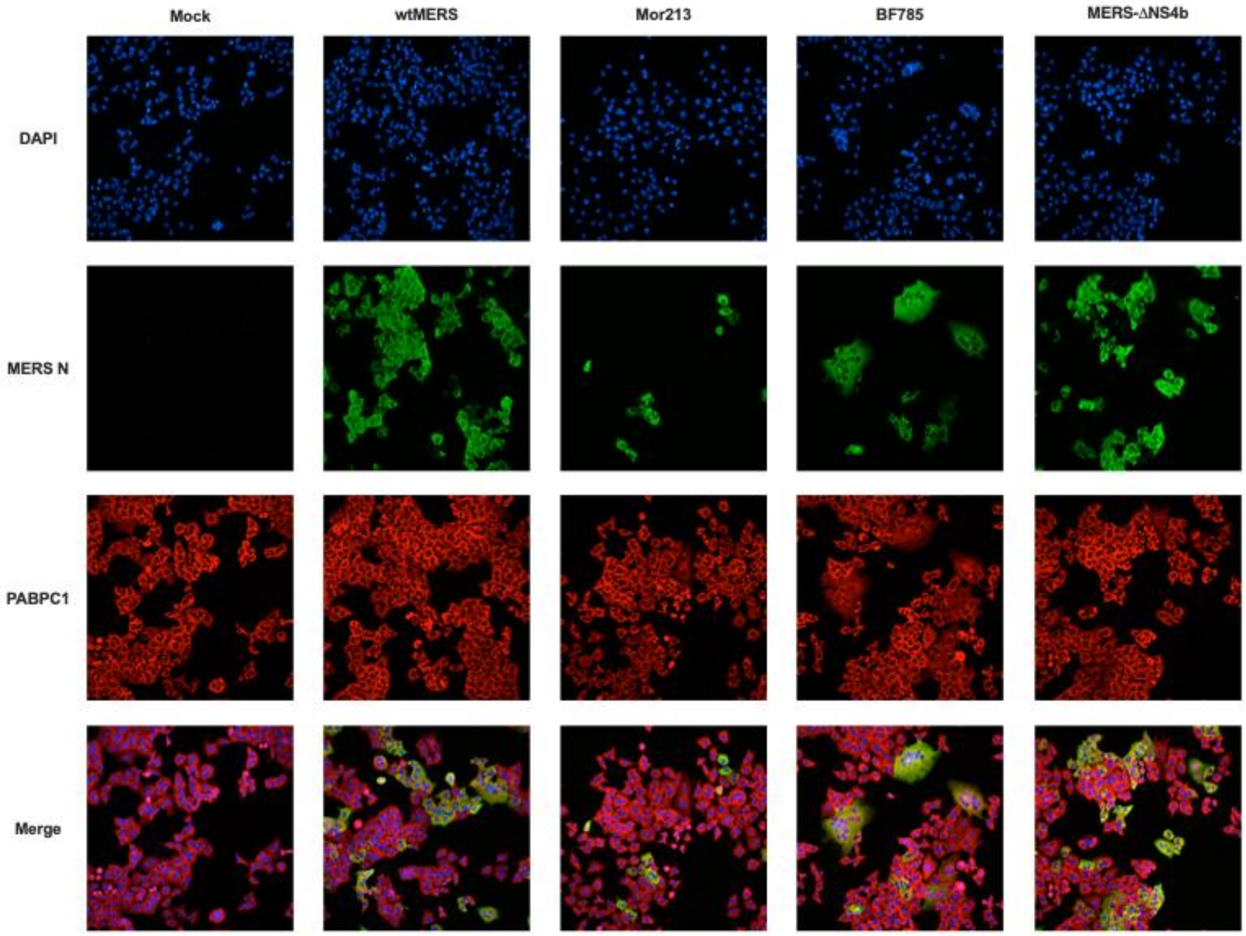

Supplementary Figure S2. A549^{DPP4} cells were infected in duplicate at MOI = 0.5 PFU/cell with wtMERS, Mor213, BF785, and MERS-ΔNS4b, fixed 24 hpi in 4% PFA, and stained for MERS N (green), PABPC1 (red) and DAPI (blue). Images were acquired on a Nikon Eclipse Ti 2. All experiments are n = 3 in technical duplicate with 6 images taken per condition per experiment.

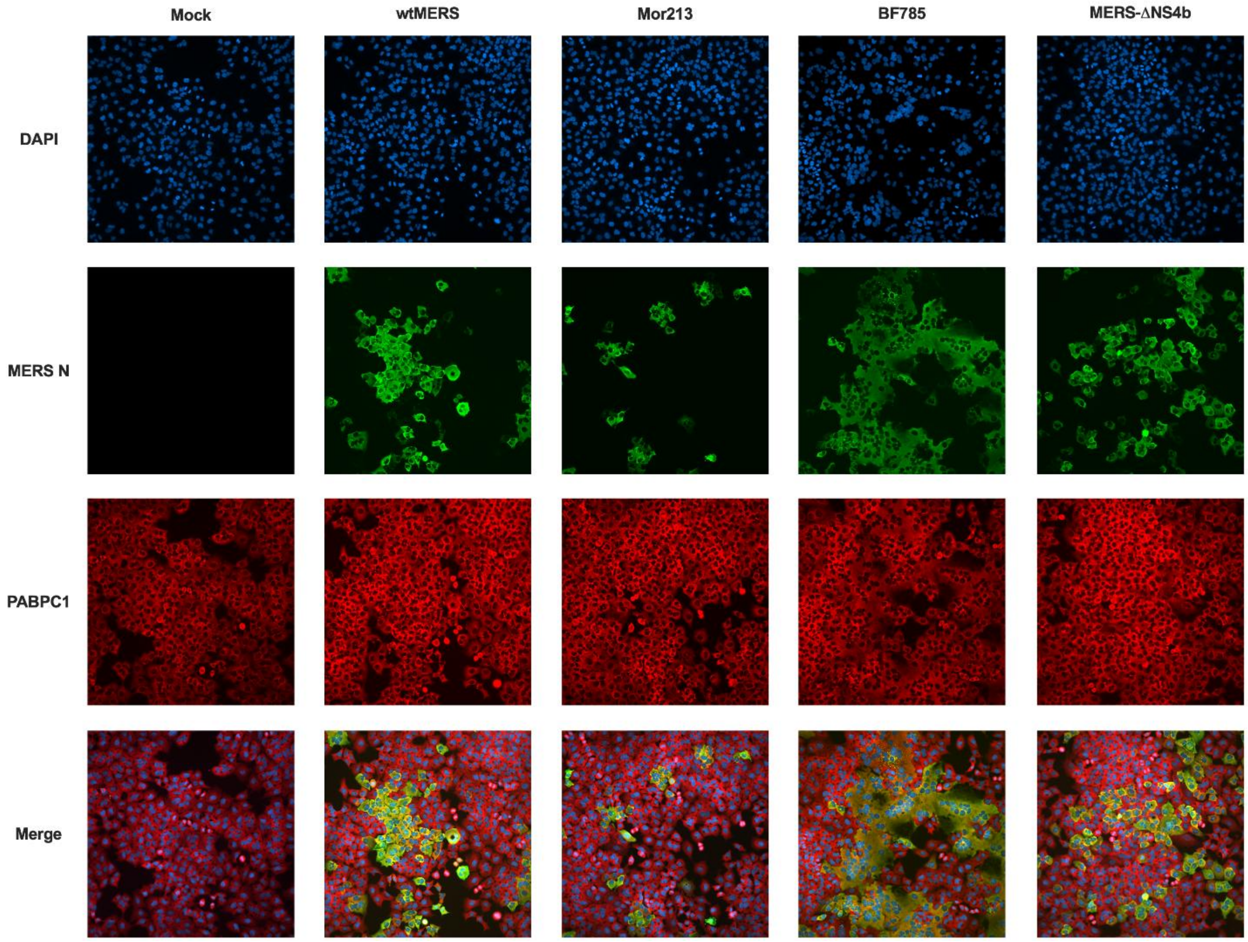

Supplementary Figure S3. A549[DPP4] *RNASEL* KO cells were infected in duplicate at MOI = 0.5 PFU/cell with wtMERS, Mor213, BF785, and MERS-ΔNS4b, fixed 24 hpi in 4% PFA, and stained for MERS N (green), PABPC1 (red) and DAPI (blue). Images were acquired on a Nikon Eclipse Ti 2. All experiments are n = 3 in technical duplicate with 6 images taken per condition per experiment.
